# Telomere Length Maintenance and Its Transcriptional Regulation in Lynch Syndrome and Sporadic Colorectal Carcinoma

**DOI:** 10.3389/fonc.2019.01172

**Published:** 2019-11-05

**Authors:** Lilit Nersisyan, Lydia Hopp, Henry Loeffler-Wirth, Jörg Galle, Markus Loeffler, Arsen Arakelyan, Hans Binder

**Affiliations:** ^1^Group of Bioinformatics, Institute of Molecular Biology, National Academy of Sciences, Yerevan, Armenia; ^2^Interdisciplinary Centre for Bioinformatics, Leipzig University, Leipzig, Germany; ^3^Institute for Medical Informatics, Statistics and Epidemiology, Leipzig University, Leipzig, Germany

**Keywords:** telomere attrition, colorectal cancer, mismatch repair, telomerase and alternative telomere maintenance, pathway models, DNAseq and RNAseq data analysis, telomere length, telomere repeat variants

## Abstract

**Background:** Activation of telomere maintenance mechanisms (TMMs) is a hallmark of most cancers, and is required to prevent genome instability and to establish cellular immortality through reconstitution of capping of chromosome ends. TMM depends on the cancer type. Comparative studies linking tumor biology and TMM have potential impact for evaluating cancer onset and development.

**Methods:** We have studied alterations of telomere length, their sequence composition and transcriptional regulation in mismatch repair deficient colorectal cancers arising in Lynch syndrome (LS-CRC) and microsatellite instable (MSI) sporadic CRC (MSI s-CRC), and for comparison, in microsatellite stable (MSS) s-CRC and in benign colon mucosa. Our study applied bioinformatics analysis of whole genome DNA and RNA sequencing data and a pathway model to study telomere length alterations and the potential effect of the “classical” telomerase (TEL-) and alternative (ALT-) TMM using transcriptomic signatures.

**Results:** We have found progressive decrease of mean telomere length in all cancer subtypes compared with reference systems. Our results support the view that telomere attrition is an early event in tumorigenesis. TMM gets activated in all tumors studied due to concerted overexpression of a large fraction of genes with direct relation to telomere function, where only a very small fraction of them showed recurrent mutations. TEL-related transcriptional state was dominating in all CRC subtypes, showing, however, subtype-specific activation patterns; while contribution of the ALT-TMM was slightly more prominent in the hypermutated MSI s-CRC and LS-CRC. TEL-TMM is mainly activated by over-expression of DKC1 and/or TERT genes and their interaction partners, where DKC1 is more prominent in MSS than in MSI s-CRC and can serve as a transcriptomic marker of TMM activity.

**Conclusions:** Our results suggest that transcriptional patterns are indicative for TMM pathway activation with subtle differences between TEL and ALT mechanisms in a CRC subtype-specific fashion. Sequencing data potentially provide a suited measure to study alterations of telomere length and of underlying transcriptional regulation. Further studies are needed to improve this method.

## Introduction

The view on telomeres has progressed from simple caps that conceal chromosome ends from DNA repair machinery (1, 2) to complex structures involving hundreds of proteins that have an active role in organizing the genome (3, 4). Telomeres are shortened with each cell division and finally trigger a DNA-damage response resulting in senescence (5). Tumors avoid this by adding newly synthesized telomeric DNA to the chromosome ends via a telomere length maintenance mechanism (TMM), which counteracts telomere shortening and saves the tumor cells from the onset of telomeric crisis thus essentially contributing to cancer progression (6). In most tumors, TMM gets activated via the telomerase pathway (TEL) which utilizes the telomerase ribonucleoprotein containing an RNA template for telomeric DNA synthesis (7). The TEL-TMM is typically active in germline, and to a less degree, in stem cells, but not in somatic cells, due to transcriptional silencing of the TERT-encoded catalytic subunit of telomerase (7, 8). A lower proportion of tumors activates an alternative lengthening of telomeres (ALT) pathway that relies on homologous recombination events between telomeric strands of sister chromatids, distant chromosomes, or extrachromosomal telomeric repeat sequences (9, 10). Usually ALT is associated with altered chromatin environment at telomeres, frequent mutations in ATRX and DAXX genes, the presence of extra-chromosomal telomeric repeat sequences and ALT-associated promyelocytic leukemia bodies (APB) (11, 12).

Most of the tumors (70–90%) are usually assumed to utilize TEL-TMM, while the rest are thought to refer to ALT-TMM (10). Several studies in the last years suggest a more diverse picture where tumors seem to characterized not by just one TEL or ALT TMM phenotype. A recent PanCancer study cross 31 tumor types demonstrated that 73% of the analyzed samples expressed TEL, 5% was associated with ALT, while the remaining 22% of tumors neither expressed clear TERT nor harbored ALT-associated alterations (13). This result is supported by reports that in a so-called ever-shorter telomeres phenotype neither of the two TMMs get activated (14). In addition to such “neither ALT nor TEL” situations, also “TEL and ALT coexistence” *in vitro* and in cancer and “TEL-to-ALT switching” situations were discussed [see (12) and references cited therein]. Mutations of ATRX and of TERT are not sufficient as possible indications for ALT- and TEL-TMM because loss of ATRX coexist with TEL-TMM in some cell lines (15) and melanomas, which can show ATRX and TERT mutations in parallel (16), while they are mutually exclusive in in glioma (17). On the other hand, TERT promoter mutations are not enough to cause activation of telomerase (18). Despite emerging conceptual models, e.g., to explain TEL-to-ALT switching in epithelial tissues (12), it remains largely unclear as to why TEL and/or ALT become activated in specific cancer subsets and what is the molecular mechanism (19).

TEL-positive tumors are typically identified by mutated and/or activated TERT where however about 20% of CRC do not show this characteristics (20). ALT-positive tumors are often deduced from the presence of telomere length maintenance in the absence of TERT activity and/or by assays based on genetic or phenotypic markers, such as the presence of C-circles and/or APBs, but these assays are potentially not definitive for several reasons (21). For example, existence of APBs does not yet ensure telomere synthesis (22). On the other hand, C-circles may be missing in cells with otherwise high ALT activity (22).

Whole genome DNA and RNA sequencing data open novel perspectives for studying telomere length dynamics and TMM in cancer. Here we have applied a bioinformatics approach of telomere length and of sequence variant computation based on DNA-seq data, where, at least the former application represents a robust and accurate alternative to experimental techniques (23–25). This structural information about telomeres is combined with a thorough expression analysis of genes contributing to TEL and ALT activation to shed light into aspects of the underlying transcriptional regulation of TMM. *Omics* data are frequently available in many molecular cancer studies and data repositories, such as The Cancer Genome Atlas (https://www.cancer.gov/about- nci/organization/ccg/ research/ structural-genomics/tcga). They offer an alternative and independent option for studying telomere biology of cancer based on omics data and judging the telomere status as a potential marker of disease development. Understanding the mechanisms regulating telomere length is of importance for development of telomere-targeted cancer therapies (26, 27) and also for identification of markers suited for characterization of early and later stages of cancer development.

TMM may vary from cancer to cancer, and even among cancer subtypes. Consequently, the study of TMM requires a tumor-type specific approach. For example, dysregulation of telomere length is a hallmark of colorectal cancer (CRC), but reports of telomere lengths and their ascribed cancer risks have been discordant, with both very short and very long telomeres implicated (28–30, 30–33). While most studies have addressed telomere length alterations in CRC (30, 32, 34), the mechanisms of telomere length maintenance regulation and, particularly, the role of mismatch repair deficiency in TMM are still not fully characterized. Here, we focus on CRC showing microsatellite instability (MSI) arising from dysfunctional mismatch repair (MMR) mechanisms in Lynch syndrome (LS-) CRC and in sporadic (s)-CRC as well. LS is one of the most frequently inherited cancer predisposition syndromes contributing to about 3% of all CRC cases (35, 36). It is defined by an autosomal dominant heterozygous constitutional mutation in one of the four key MMR genes MLH1 (about 60%), MSH2 (about 30%), MSH6 or PMS2 (37, 38) all leading to MSI. In contrast, MSI in s-CRC most frequently results from promoter hyper-methylation of the MLH1 gene giving rise to about 20% of all CRC cases (39, 40). The MMR machinery not only has a role in mismatch repair, but also in cell cycle checkpoint activation and DNA damage induced cell cycle regulation. Proteins involved in the MMR pathway, such as PCNA, RPA, and DNA polymerase δ, are also important players in ALT-TMM (30, 41). It has been reported that MSH2 deficiency can accelerate telomere shortening (42). Additionally, it has been shown that MSH6-MMR deficiency leads to a hyper-recombinant phenotype, increased survival of tumor cells in response to telomerase inhibition and shows some evidence of telomeric sister chromatid exchange that are possible signs of ALT (43). Another study has observed a trend of lower expression of TERT and high levels of APBs in MMR-deficient gastric cancer (44). However, possible activation of the ALT TMM in response to MMR-deficiency in CRC still has to be investigated.

With this aim our study addresses TMM of MSI cancers in LS-CRC and in s-CRC, and also in benign colon mucosa and in MS stable (MSS) s-CRC for comparison, which overall constitutes about 60% of all CRC cases. Our study is based on whole genome DNA and RNA sequencing data of patient matched tumor and tumor-distant mucosa samples generated recently by us (45) and of s-CRC data taken from the TCGA repository (40). An interesting aspect results from the fact that cancerogenesis of LS-CRC is driven by immune escape from inflamed non-cancerogenous mucosa (36, 46) with possible impact on telomere biology. The publication is organized as follows: in the first part we analyze alterations of telomere length and of the abundance of canonical and non-canonical telomere repeat variants in the different tumor subtypes and in the reference mucosa systems. In the second part we study how TEL and ALT TMM are regulated at transcriptional level, thus forming different TMM phenotypes.

## Materials and Methods

### DNA- and RNA-seq Data

We made use of whole-genome DNA-seq and RNA-seq data of Lynch Syndrome (LS) referring to paired patient-matched fresh frozen tissue specimens of tumor and tumor-distant non-neoplastic mucosa (reference samples), which were collected from 11 LS-CRC patients, as described and characterized in Binder et al. (45). Tumor samples split into adenoma (*N* = 3) and cancer (*N* = 9) specimen with only one patient-matched adenoma-cancer pair (samples were assigned by patient no. and “reference,” “adenoma” or “cancer” sample types). DNA- and RNA-seq data refer to the same mucosa and tumor samples. The data are available at the dbGaP database (www.ncbi.nlm.nih.gov/gap) under accession number phs001407). According to our previous analysis, the LS cases split into two genetically distinct groups named G1 (six patients) and G2 (five patients). G1 tumors showed higher load of somatic mutations (108.000 vs. 34.000 per tumor), a higher number of MLH1 constitutional mutations (5x MLH1 and 1x MSH2 vs. 1x MLH1, 2x MSH2 and 1x MSH6) and higher microsatellite slippage rate, compared to G2 (45). For comparison, we included sequencing data of microsatellite stable (MSS) and instable (MSI) sporadic CRC (s-CRC) cases and of healthy (normal) colonic mucosa taken from the TCGA repository as described in Binder et al. (45). DNA-seq data were taken from patient matched pairs of s-CRC tumors and normal mucosa (5 MSS cases and 8 MSI cases). RNA-seq data refer to unmatched cases of reference mucosa (20 samples), MSS s-CRC (21), MSI-low s-CRC (24), and MSI-high s-CRC (20). In accordance with previous studies (47) the MSS and MSI-low samples were subsumed into one combined MSS group. In support of this, transcriptome patterns along the chromosomes show clearly a common chromosome instability phenotype for MSS and MSI-low s-CRC in contrast to MSI-high s-CRC samples (48), which were assigned the CpG hypermethylation phenotype (CIMP, Supplementary Figure 1). MSI-high cases were annotated as MSI throughout the paper. TCGA-accession numbers of all cases studied were listed in Supplementary Table 2 in Binder et al. (45).

### Telomere Length and Telomeric Repeat Variants

Mean telomere lengths (MTL) were calculated using the whole genome DNA-seq data and the program Computel (v1.2, accessible at: https://github.com/lilit-nersisyan/computel) using default parameter settings (25). This program detects reads originating from telomeres by alignment to a reference sequence that consists of telomeric repeat patterns (25). It then computes MTL across the chromosomes in units of base pairs (bp), by comparing the coverage at the telomeric reference to the total sequencing depth and normalizing to the number of chromosomes. All LS-tumors, and all s-CRC tumors, except for one, were diploid [see Supplementary Table 1 in Binder et al. (45) which also provided detailed sample characteristics in terms of constitutional mutations, microsatellite status, tumor cell content and patient characteristics, and (49) for s-CRC]. Among s-CRC MTLs were computed for all the runs per sample, and the median MTL was taken for subsequent analysis. Computel also estimates the composition of telomeric repeat variants (TRVs), providing the amount of canonical (“TTAGGG”) and non-canoncial TRVs. In contrast to pattern matching algorithms, Computel is not restricted to predefined non-canonical variants, but can capture any variation, be it substitution, insertion or deletion.

### Gene Expression Analysis

Identification of differentially expressed genes (DEGs) was performed based on read count data using Wald test implemented in DESeq2 package (50). For functional interpretation of gene expression data we applied gene set analysis in terms of gene set enrichment z-score (GSZ) profiles (51). Gene sets were taken from the GSEA-repository and from literature for different functional categories (52).

### Pathway and Network Analysis of Telomere Maintenance Mechanisms

The genes and pathways involved in TEL and ALT TMM were taken from a literature search and pathway reconstruction approach using reference gene expression data in TEL- and ALT-positive cell systems [see (53, 54) and Supplementary Methods for details]. A list of TMM genes is provided in Supplementary Table 1 together with two independent verifications by means of enrichment analysis in gene ontology categories (Supplementary Table 2) and their characteristics as provided by TELNet telomere knowledge base (Supplementary Table 3). The activity of the TMM-pathways was estimated by means of the pathway signal flow (PSF) algorithm (55) using the TMM app for Cytoscape. It estimates the transcriptional activity of each pathway node in terms of PSF-scores making use of the local pathway topology and of gene expression fold changes compared to average expression as described in Nersisyan et al. (55, 56). The impact and specifics of PSF-pathway analyses compared with gene set approaches were demonstrated recently in a series of applications to characterize aberrant pathway activation in the context of different diseases (45, 57–59).

We performed TMM-based computations for each of the LS- and s-CRC groups separately. The PSF scores of the different TEL and ALT pathway branches and of the final sink nodes were used to characterize the different tumor subtypes. To estimate the effect, which a selected gene exerts on a certain node of the pathway, we have calculated the partial influence (PI)-score. It is defined as the node's differential PSF-score upon neutralizing the affecting gene by setting its expression fold change to unity. We used the PI-score to select the genes that exert strongest effect on the PSF-scores of the major TMM-branches, either as activators (PI > 0) or as inhibitors (PI < 0), with respect to mean pathway activity of the respective group of samples (see also Supplementary Figure 2).

The correlation networks of gene expression and PSF values of the TMM network nodes were constructed using a Pearson correlation significance threshold of *p* < 0.05 for edge selection. Visualization and betweenness centrality (BC) analysis were performed with NetworkAnalyzer in Cytoscape 3.6 (60).

## Results

### Telomeres Predominantly Shorten in CRC as an Early Event in Tumor Development

**Figure 1:**
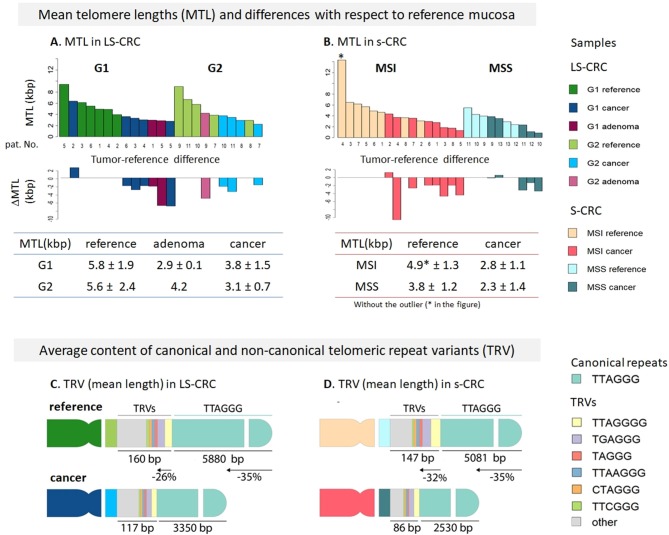
Mean telomere length (MTL) and telomere repeat variant (TRV) analysis in Lynch syndrome and sporadic colorectal cancer. MTL and its differences in tumors with respect to paired reference mucosa samples for LS-CRC **(A)** and s-CRC **(B)** indicate that telomeres broadly get shorter in all tumor types on the average (see Supplementary Table 4A for details). Average TRV content in reference and tumor samples of LS-CRC **(C)** and s-CRC **(D)** showed that non-canonical repeats get shorter at slightly lower rates (26–32%) compared to canonical repeats (35%) which suggests their accumulation in the sub-telomeric region as indicated schematically in the figure. The TRVs comprise only 1–2% of the telomere length on the average. The TRV shortening showed a consistent trend in all samples (see Supplementary Figures S1, S2).

In order to explore telomere length changes during malignant transformations, we have analyzed mean telomere length (MTL) in LS-CRC and in s-CRC from whole genome sequencing data using Computel software (25). MTL systematically shortens in all tumor tissues of types G1 and G2 LS-CRC and in MSI and MSS subtypes of s-CRC compared to the respective reference mucosa samples (Figures 1A,B), which is in agreement with prior knowledge (56). On average, MTL decreases by 2.7 and 2.3 kbp in G1 and G2 LS-CRC, by 2.7 kbp in MSI s-CRC and only by 1 kbp MSS s-CRC (see also Supplementary Table 4A). The larger differences in LS-CRC and MSI s-CRC are in agreement with previous observations that link MSI and (sporadic) defects in MMR with higher telomere shortening rates (31). The MTL-differences between the cancer subtypes and the respective reference mucosa can be eventually attributed to different mean ages of the respective patients (44 ± 9 vs. 53 ± 15 years for G1 and G2 LS-CRC patients, respectively; and 63 ± 12 vs. 75 ± 12 years for MSI and MSS s-CRC, respectively) and the overall age-related shortening of telomeres in healthy colon mucosa (30, 61), and eventually also CRC (62), which suggests shorter telomeres in the mucosa of older patients (see also Supplementary Figure 3 for detailed analysis). Overall, we find a broad decrease of mean telomere length in all cancer subtypes.

### Telomeric Repeat Variants Suggests Accumulation Near Proximal Regions Without Substantial Changes of Their Composition

Telomeres are not merely composed of canonical TTAGGG repeats, but can also incorporate several types of repeat variants (TRV), such as TCAGGG, TGAGGG, and GTAGGG, particularly in the proximal telomeric and subtelomeric regions (63–65). In order to estimate whether novel TRVs are generated during malignant transformations or as a result of dysfunctional mismatch repair machinery, we have computed the TRV content in our samples. Figures 1C,D schematically depicts the average changes in TRV content (mean length in units of bp) in LS-CRC and s-CRC cancers and in reference mucosa. All the samples showed similar TRV distributions (Supplementary Figures 4–6). In LS-CRC and s-CRC, the most abundant non-canonical repeat variants all terminated with “GGG,” in agreement with the notion of strong selective pressure of this sequence (63). The top TRVs were the G- and A-insertion variants TTAGGGG and TTAAGGG, the (TG)-substitution variant TGAGGG and the T- and A-deletion variants TAGGG and TTGGG, respectively (Supplementary Figures 4–6). The mean cumulative length of the TRV was within the range of 20–60 bp per chromosome end, which, in total, comprises <1% of the overall MTL. The shortening rate of canonical TTAGGG repeats (35% in LS-CRC and s-CRC) was slightly higher compared to non-canonical TRVs (26% in LS-CRC and 32% in s-CRC). This difference can be explained by a biased placement of non-canonical TRVs toward the proximal (centromeric) regions of telomeres (Figures 1C,D). Further differences are noted when comparing TRV in MSI vs. MSS s-CRC. The mean length of TRVs was larger in MSI, consistent with longer telomeres in this subtype (Figure 1). Concomitantly, the percentage of most TRVs was lower in MSI tumors, as well as in reference samples compared to MSS (Supplementary Figure 6). Relative lower proportion of TRVs were previously reported in ALT positive vs. ALT negative cancers, also attributed to longer telomeres in the former (66). Interestingly, selected TRVs such as the C-substitution variants TTCGGG and TCAGGG are found to show largest differential lengths in our data (Supplementary Table 3B). TRV analyses largely suggests a small effect size and their likely accumulation in proximal telomeric regions, with selected TRVs (e.g., TTCGGG), showing different trends compared to the rest of the TRVs (66).

All in all, the effects we have observed are small in amplitude and mechanistically not fully understood. Additionally, we also find similar differences in the reference system of MSI and MSS s-CRC. Therefore, TRV dynamics require further, more systematic studies.

### TMMs Compensate for Proliferative Telomere Attrition

**Figure 2:**
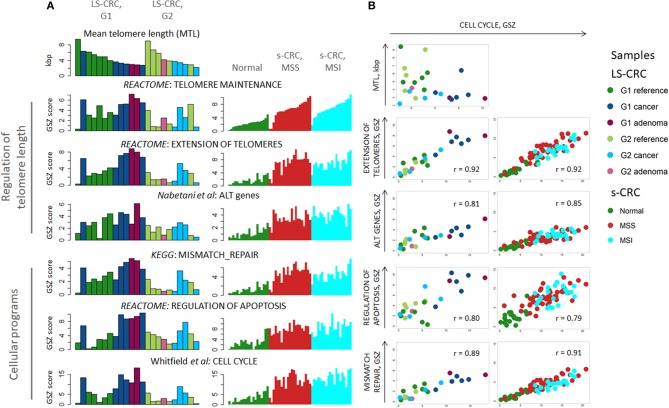
Transcriptome analysis of cellular programs associated with regulation of telomere lengths: **(A)** The gene-set Z-score (GSZ) profiles reflect activation of cellular programs ensuring lengthening of telomeres, cell division, apoptosis and DNA mismatch repair in LS-tumors and s-CRC compared with reference mucosa. LS-CRC samples are sorted with decreasing telomere length in each sample group (**A**, top-left), while s-CRC samples are ranked with increasing GSZ-score of the gene set “telomere maintenance” because of lack of MTL-information. **(B)** Biplots of the GSZ-scores of the gene sets “extension of telomeres,” “ALT genes,” “mismatch repair” and “regulation of apoptosis” as a function of cell cycle activity suggest a high degree of co-regulation. Note that there is virtually no gene overlap between the gene sets. The plot of MTL as a function of cell cycle activity indicates that telomere lengths asymptotically levels off toward a lower critical MTL-limit in the tumors with increasing cell cycle activity. This trend reflects the fact that replicative telomere loss in tumors is compensated by upregulation of “telomere length maintenance” and “extension of telomeres” mechanisms.

We next proceeded with gene set analysis to identify biological processes associated with telomere length regulation. We considered two Reactome gene sets for telomerase-based elongation of telomeres (“extension of telomeres” and “telomere maintenance”) and one gene set related to alternative lengthening mechanism collecting genes involved in ALT obtained from literature (67). Since activation of TMM usually accompanies the processes of apoptosis and DNA damage-response in most cancer cells, we have also analyzed cellular programs related to cell division, namely, KEGG “mismatch repair”, Reactome “regulation of apoptosis” and “cell cycle” taken from Whitfield et al. (68) (Figure 2A). They are clearly at lower activity levels in G2 LS-CRC compared to G1, even though MTL shortening is comparable in both subtypes (Figure 2A). Possible reasons of this difference between G1 and G2 are addressed below. The comparison of TMM gene sets between tumor and reference tissue of each LS-CRC subtype showed that the telomerase based TMM is markedly activated both in G1 and in G2 cancers, while the ALT-TMM shows, if at all, only weak activation in tumors. Similar to LS-CRC, the TMM- and the cell division-related gene sets show transcriptional activation in MSS and MSI s-CRC compared to normal mucosa. We also observe activation of the ALT gene set in the MSI and, to a slightly smaller degree, in the MSS s-CRC subtypes.

Plots combining the GSZ-scores of the gene sets with that of cell-cycle activity show marked correlation in all cases, which suggests a high degree of mutual co-regulation, particularly between cell cycle on one hand and TMM, apoptosis and MMR on the other hand (Figure 2B). In other words, high cell cycle rates obviously require also high rates of MMR and of TMM to compensate for replication errors and telomere attrition, respectively, which, in turn, relate to increased apoptosis rates (69) that require feedback toward increased cell cycle activity for net survival of the cells. On one hand, TMM, especially TEL, represses apoptosis via telomere maintenance and probably also by extra-telomeric functions of *TERT*, e.g., via modulation of oxidative stress in mitochondria and interactions with apoptotic pathways [see (70) and references cited therein]. On the other hand, only a part of cells acquires immortality at telomere crisis and proceeds to cancerogenesis while the other part becomes apoptotic (71). Our transcriptomics data thus suggest a direct relation between cell cycle, TMM and apoptotic regulation rates. Note also that the data points of MSI s-CRC are systematically shifted toward smaller values for “extension of telomeres” and “mismatch repair” compared with MSS s-CRC, which reflects lower activity of these processes in MSI s-CRC at the same proliferation rate. This kind of feedback is also observed in reference mucosa, which means that the feedback mechanism is obviously not restricted to tumors, but is also present in pre-neoplastic reference mucosa. Hence, TMM seems to follow rather a continuous than a stepwise activation beyond a certain threshold. This hypothesis is further supported by the plot of the MTL of the LS samples as a function of cell cycle activity. It demonstrates that MTL decays non-linearly with increased proliferation rate and levels off into a lower critical value in tumors (Figure 2B, part top-left). In other words, telomere attrition due to increased cell cycle activity in tumors gets compensated by TMM resulting in a low, “steady state” critical MTL-value. Overall, we find that a whole battery of cellular processes must get up-regulated in concert with cell division rates in order to maintain proper cell functionality, and particularly, a minimum critical telomere length required for cell survival.

Concerted activation of TMM, mismatch-repair, cell cycle and apoptosis related gene sets in cells with high proliferative activity inherently imply that unsupervised analyses of gene expression, e.g., based on correlation with MTL, usually reveal not only canonical TMM genes, but also a large number of genes involved in other cellular programs. To avoid these interferences of mostly unknown background, we focus on a set of genes involved in TMM pathways which have previously been selected based on literature reports and reference gene expression data (53).

### Telomerase (TEL) and Alternative (ALT) TMM Pathways in LS-CRC and s-CRC

**Figure 3:**
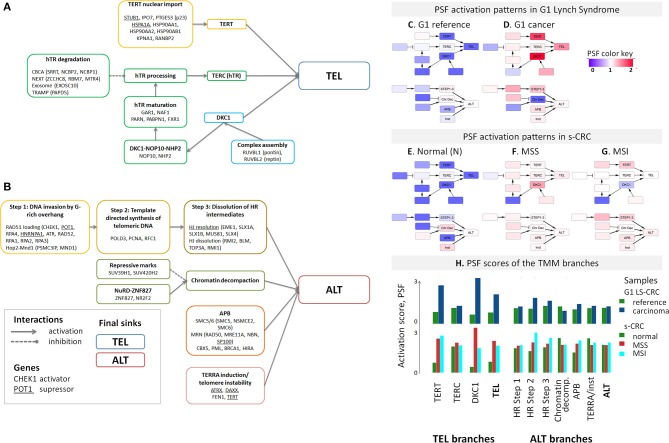
Schematic representation of the TEL **(A)** and ALT **(B)** TMM pathways and their mean PSF-activation patterns averaged over the tumors of each CRC subtype **(C–H)**. The most relevant genes acting either as activators or suppressors are listed in each of the nodes [see (53) for details]. The color of the nodes in part **(A,B)** codes the respective genes and processes throughout the paper. The TEL and ALT-TMM get activated in all CRC subtypes compared with reference mucosa. **(H)** The barplot of the PSF scores of the major TMM-pathway branches reveal that TEL pathway activation in G1 LS-CRC occurs mainly through TERT and DKC1 branches. In s-CRC the TEL pathway is activated either through the DKC1 and TERT branches (MSS) or merely the TERT branch (MSI). ALT-TMM activation occurs mainly via HR- Step 2 and HR-Step 3 and APB nodes in all tumor subtypes, with pronounced activation of Step 2 in MSI s-CRC and G1 LS-CRC.

For detailed supervised analysis on telomere maintenance mechanisms, we make use of previously constructed TMM pathways describing (i) the “classical” TMM that is governed by the catalytic action of the telomerase enzyme (TEL), and (ii) the alternative TMM (ALT) which is realized through homologous recombination events [Supplementary Figure 7, (53) and references cited therein]. These pathways decompose into sub-processes that concertedly affect the activity of the TEL- or ALT-TMMs (Figures 3A,B). Particularly, the final sink of the TEL-pathway collects activities from the three pathway branches related to telomerase complex components hTERT, hTR, and dyskerin, encoded by *TERT, TERC*, and *DKC1*, respectively, and processes leading to their activation, such as nuclear localization and complex assembly (Figure 3A). The ALT pathway gets activated via homologous recombination (HR) events involved in break induced repair (BIR) at telomeres, as well as by chromatin decompaction near the telomeres, accumulation of other proteins involved in ALT associated promyelocytic leukemia body (APB) formation and by TERRA induction and telomeric instability. Verification of pathway genes selected using independent knowledge information confirms enrichment of genes with direct involvement in telomere biology (see Supplementary Tables 2, 3 for details).

**Figure 4:**
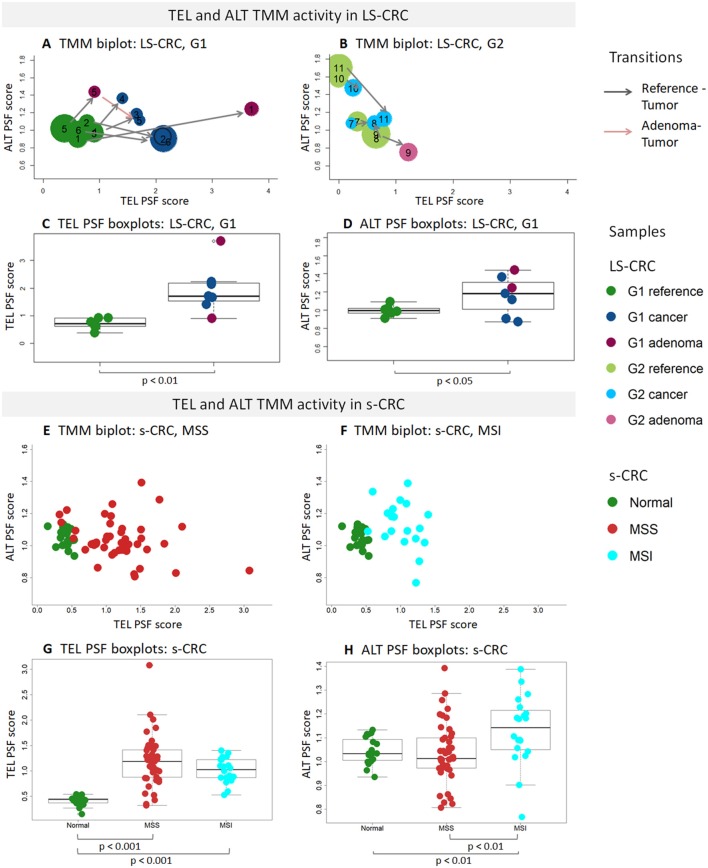
PSF analysis of TEL and ALT TMM pathways in LS-CRC, s-CRC, and the respective reference mucosa samples. The biplots show mutual activation pattern of the ALT and TEL TMM pathways in the LS-CRC **(A,B)** and s-CRC **(E,F)** samples studied. The arrows point from the reference to the tumor for the patient-matched sample pairs. They indicate consistent activation of TMM in all G1 samples but not in G2. The point sizes in **(A,B)** scale proportional to the MTL-values in LS-CRC revealing that shortening of telomeres associates with TMM activation in G1 tumors. The boxplots of the TEL **(C,G)** and ALT PSF **(D,H)** scores show activation of TEL TMM in G1 LS-CRC **(C)** and both s-CRC subtypes **(G)** on the average while ALT-TMM specifically activates in MSI s-CRC **(H)** and to a less extend in G1 LS-CRC **(D)**. Note the larger variability of ALT-TMM PSF in MSI s-CRC compared with MSS s-CRC **(H)**.

The activity of these pathways was estimated with the pathway signal flow (PSF) algorithm (53, 55, 56). The algorithm considers expression values of the genes and their mutual interactions to estimate the pathway activity in terms of PSF-scores in each the individual sample, as well as PSF-activities of each individual pathway node. We find marked activation of the TEL- and ALT- TMM pathways in G1 LS-CRC and s-CRC compared with the respective reference mucosa for each of cancer subtypes studied (Figures 3C–H). The PSF-scores of the final sinks of the TEL- and ALT-TMM pathways increase in patient-matched tumor samples compared with reference mucosa in G1 (Figures 4A,C,D), but not in G2 LS-CRC (Figure 4B). Further analysis showed that neither of the TMM genes is significantly differentially expressed in G2 tumors with respect to reference mucosa (Supplementary Figure 8B). Moreover, the G2 tumors showed relatively low cell cycle activity compared with G1 tumors (Figure 2A). Because of these facts we, excluded G2 data from further analysis, as their transcriptomes seem not to reflect the TMM phenotype of G2 cancer cells. One reason for this problem can be seen in the fact that stromal components in G2 LS-CRC samples (45) can dominate over more subtle expression traits inherent to cancer cells (72, 73).

TMM analysis of the s-CRC samples indicate considerable activation of the TEL pathway in MSS and MSI s-CRC compared to normal mucosa, while ALT-TMM gets activated specifically in MSI s-CRC (*p* = 0.004, Mann-Whitney *U* test, Figures 4E,F). Notably, MSI s-CRC show low variance of TEL pathway activity compared to MSS (*F* test *p* = 0.001), suggesting existence of a regulatory mechanism dumping variability of TEL TMM activity in these samples (vide infra). Overall, supervised TMM pathway analysis reveals pronounced activation of TEL-TMM in all cancers. Moreover, it suggests specific activation of ALT-TMM in MSI s-CRC.

### Transcriptional and Mutational Patterns of TMM Genes

**Figure 5:**
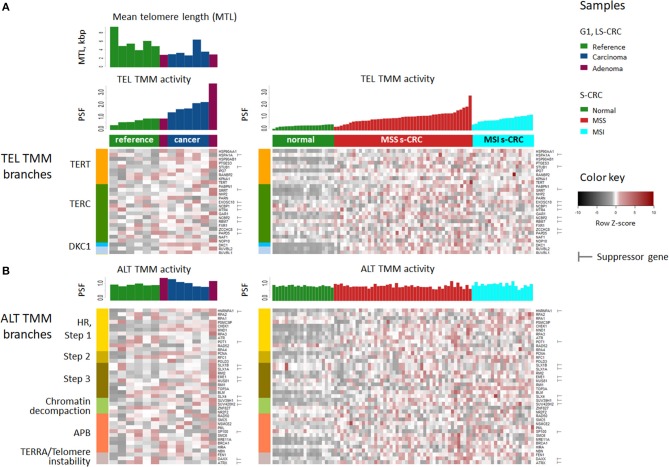
Heatmap of TMM gene expression of G1 LS-CRC (left) and s-CRC (right). The samples are ranked within each subtype with increasing PSF score of TEL TMM pathway activity shown as barplots together with the MTL **(A)** and the ALT-PSF score above the heatmap **(B)**. The genes in the heatmap are sorted according to the pathway branches they belong to. The majority of genes in all TMM branches get upregulated in CRC.

**Figure 6:**
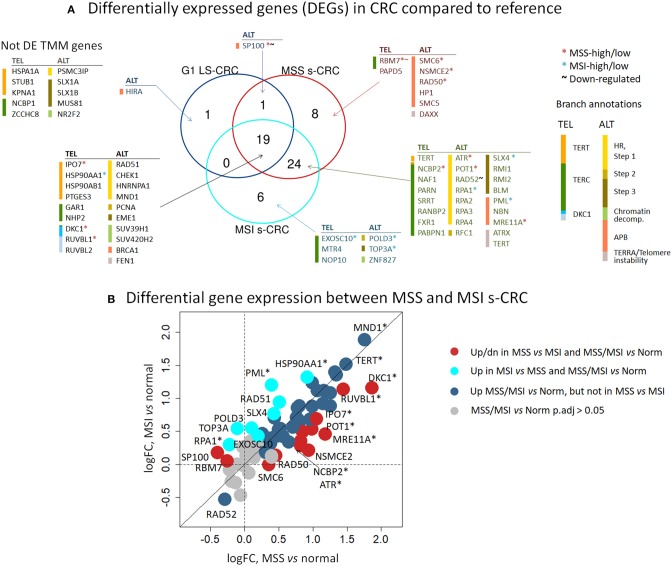
Differential expression analysis of TMM genes of tumor-vs. -reference comparison in LS-CRC and s-CRC and of MSI-vs. -MSS comparison in s-CRC. **(A)** The Venn diagram indicates that 19 out of 59 TMM genes (32%) are commonly differentially expressed between tumors of all subtypes and reference mucosa (adjusted *p* < 0.05, Wald test) and another 24 DEGs (40%) overlap between MSI and MSS s-CRC. **(B)** The biplot of the logged fold expression changes (log FC) between the tumors and reference mucosa of MSS and MSI s-CRC provides a more focused view on gene expression differences only between s-CRC subtypes and shows two types of DEGs which are differentially expressed in tumors-vs. -reference a) but not between MSS and MSI s-CRC (blue circles), or b) also between both s-CRC subtypes in either direction (red and cyan circles). DEGs with adjusted *p* smaller than 0.05 (Wald test) are indicated by asterisks.

An expression heatmap of the TMM genes, provided in Figure 5, suggests their widespread activation in cancer compared to reference mucosa. Indeed, 34% (LS-CRC) and 79% (s-CRC) of all 67 TMM genes in the TEL and ALT-pathways show significant up-regulation (adjusted *p* <0.05), while only three genes (*RBM7, SP100*, and *RAD52*) get significantly down-regulated in at least one of the subtypes (Figure 6, Supplementary Figure 9). Overall 19 TMM genes (32%) were commonly up-regulated in all three cancer types and another 24 (40%) in MSS and MSI s-CRC (Figure 6A) (see Table 1 for top genes). No gene is found down-regulated in all three cancer subtypes at once: SP100 loses expression in LS-CRC and MSS s-CRC, while RAD52 deactivates in MSS and MSI s-CRC.

**Table 1:** Top TMM genes in LS- and s-CRC according to different measures^a^.

**Method**	**TMM**	**G1 LS-CRC**		**MSS s-CRC**		**MSI s-CRC**	
**DE^**b**^**		**Gene**	**log2 FC**	**Gene**	**log2 FC**	**Gene**	**log2 FC**
	TEL	NHP2	1.92	**DKC1**	1.86	**HSP90AB1**	1.08
		**RUVBL2**^**e**^	1.74	RUVBL1	1.44	HSP90AA1	1.32
		**DKC1**	1.21	**HSP90AB1**	1.26	**DKC1**	1.16
		GAR1	1.56	IPO7	1.05	RUVBL1	1.14
		**HSP90AB1**	0.86	**RUVBL2**	0.99	**RUVBL2**	0.88
	ALT	SP100	−1.58	MRE11A	1.18	**CHEK1**	1.40
		**CHEK1**	1.75	**CHEK1**	1.32	**MND1**	1.89
		EME1	2.06	ATR	0.82	FEN1	1.24
		SUV420H2	1.31	**MND1**	1.76	BRCA1	1.36
		**MND1**	2.48	BRCA1	1.34	PML	1.20
**PI**^**c**^		**Gene**	**Mean PI**	**Gene**	**Mean PI**	**Gene**	**Mean PI**
	TEL	**TERT**	0.20	**TERT**	0.20	**TERT**	0.20
		**DKC1**	0.19	**DKC1**	0.14	**DKC1**	0.13
		**RUVBL2**	0.16	**RUVBL1**	0.06	**RUVBL1**	0.05
		**RUVBL1**	0.07	**RUVBL2**	0.05	**RUVBL2**	0.05
		GAR1	0.05	HSPA1A	−0.02	HSPA1A	−0.02
	ALT	EME1	−0.04	SUV39H1	−0.03	SUV39H1	−0.03
		**ATRX**	−0.04	**NR2F2**	0.03	**NR2F2**	0.03
		SLX4	−0.03	SUV420H2	−0.02	SUV420H2	−0.03
		**NR2F2**	0.02	**ATRX**	−0.02	PML	0.02
		BRCA1	0.02	BRCA1	0.02	**ATRX**	−0.02
**BC**^**d**^		**Gene**	**BC**	**Gene**	**BC**	**Gene**	**BC**
	TEL	-	-	DKC1	197	PTGES3	252
		-	-	RUVBL2	121	**TERT**	125
		-	-	**TERT**	121	NAF1	59
		-	-	RANBP2	120	PARN	56
		-	-	SRRT	117	HSP90AA1	54
	ALT	-	-	BLM	178	EME1	352
		-	-	FEN1	111	ATRX	330
		-	-	MND1	101	HNRNPA1	194
		-	-	BRCA1	92	RPA3	193
		-	-	ATR	84	NR2F2	176

a *The full list of all TMM genes with differential expression values is given in Supplementary Table 1*.

b *Mean differential expression (DE) of genes in CRC vs. reference (log2 fold change (FC), adjusted p < 0.001, Wald test)*.

c *Partial influence (PI) of genes on TEL and ALT pathways averaged over sample groups*.

d *Betweenness centrality (BC) of genes in the pairwise gene expression correlation network in MSS and MSI s-CRC. Not computed for LS-CRC, because of small sample size*.

e *Redundantly found genes were highlighted in bold font*.

Analysis of somatic mutations of the tumors of all three types doesn't reveal high mutational recurrence of TMM genes and also no clear effect of mutations on gene expression in G1 LS-CRC (Supplementary Figure 10). Interestingly, we found four genes (*FXR1, RAD50, SP100, SMC6*) mutated in 50% of the G1 LS-cancer samples, with the latter three belonging to the APB branch of the ALT-TMM pathway. All four genes are also recurrently mutated in MSI s-CRC in more than 40% of cases what suggests eventually a mutation-driven mechanism of activation of the APB-branch in G1 LS- and MSI s-CRC as well. No recurrently mutated TMM genes were found in MSS s-CRC possibly due to smaller mutational load compared with the hypermutated subtypes LS-CRC and MSI s-CRC. Besides mutations, epi-mutations, via, e.g., alterations of DNA-methylation patterns in the promoter regions of the genes can affect their expression level. CIMP gene signatures obtained from independent MSI s-CRC and LS-CRC datasets don't show pronounced differential methylation in the promoter regions of TMM genes which makes DNA methylation, at least not a dominant factor that shapes TMM activity (Supplementary Figure 1).

In summary, TMM gets activated in all cancers studied due to concerted overexpression of a large fraction of the TMM genes, which seems not to be driven by mutations and/or aberrant DNA-methylation of these genes. In LS-CRC and MSI s-CRC recurrent mutations were found in a few genes of the APB branch of the ALT pathway.

### *TERT* and *DKC1* Activate TEL-TMM

Genes of the DKC1 and TERT branches of the TEL-TMM were commonly up-regulated in all three cancer types (Figure 6A), which resulted in the markedly increased PSF-score along these pathway branches (Figures 3C–H). The TERT branch involves expression of *TERT*, the catalytic subunit of telomerase, as well as factors supporting and repressing its posttranslational activation (53, 74–76). Activating genes in this branch, first of all *TERT*, heat shock protein 90 (*HSP90AA1, HSP90AB1*), importin 7 (*IPO7*) and p23 (*PTGES3*) are overexpressed in cancer, while the heat shock protein 70 (*HSPA1A*) and CHIP ubiquitin ligase (*STUB1*), both acting as suppressors, are underexpressed (Figure 5). Note that *TERT* gets up-regulated in MSI and MSS s-CRCs as well (adjusted *p* < 0.05, Figure 6A). In G1 LS-CRC, it is not among the top up-regulated DEGs (adjusted *p* = 0.23), however it shows highly variant response with strong activation in two-three patients and weak activation in four out of seven tumors (Supplementary Figures 8, 9, Supplementary Table 5).

The genes of the DKC1 branch including *DKC1*, encoding the telomerase subunit dyskerin, and the telomerase complex assembly genes Pontin (*RUVBL1*) and Reptin (*RUVBL2*) are consistently up-regulated in all cancer subtypes (Figures 5, 6, Supplementary Figure 9). Expression of *RUVBL1* and *DKC1* progressively increases with telomere length in G1 LS- mucosa (see the plots for these genes in Supplementary Figure 11). Also, previous studies report overexpression of *DKC1* upon telomere shortening (77) and increased proliferation (78). The overexpression of *DKC1* and *RUVBL1* in s-CRC is more prominent in MSS, than in MSI (adjusted *p* < 0.05, Figure 6B), which explains the less pronounced activation of the DKC1 branch in MSI s-CRC (Figure 3H) and presumably also the lower variability of TEL activity in MSI compared to MSS (Figure 4).

**Figure 7:**
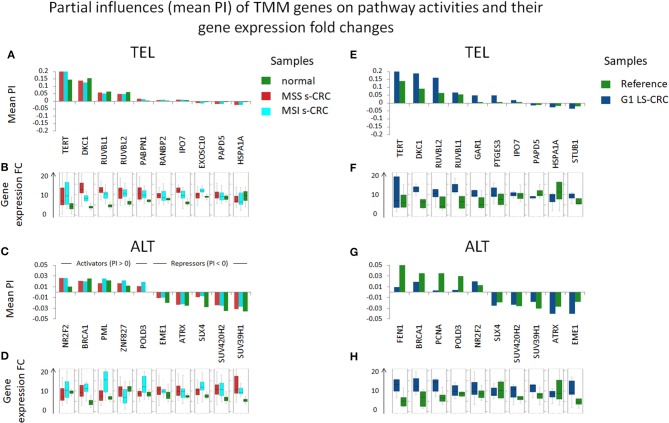
Partial influences (PI) of TMM genes on TEL and ALT pathway activities and their expression differences in units of log FC. *DKC1* and *TERT*, as well as *RUVBL1* play strongest roles in TEL pathway activity in all cancer subtypes **(A,E)**. The ALT TMM pathway shows less pronounced PI amplitudes: it becomes activated by *NR2F2* and *BRCA1* and suppressed by chromatin modifiers (*SUV420H2, SUV39H1*), holiday junction proteins (*SLX4, EME1*) and *ATRX* **(C,G)**. These observations are mostly (with some exceptions) supported by respective gene expression changes **(B,D,F,H)**.

Next, we evaluated the gene's partial influence (PI) on pathway and branch activity. We find that *TERT* and *DKC1* are indeed the most influential genes strongly affecting the activity of the TEL sink in all CRC subtypes (Figure 7). *RUVBL1* and *RUVBL2* are among the top four genes influencing the ALT sink, with a prominent effect of *RUVBL2* specifically in LS-CRC. At branch level, we observe subtype-specific differences (Supplementary Figures 12, 13). In particular, the TERT branch is affected by p21 (*PTGES3*) in LS-CRC, while in s-CRC cancers, we observe high influence of heat shock proteins (*HSP90AB1* and *HSPA1A*). In MSI s-CRC, the TERT branch is activated by *RANBP2*, a gene encoding a nuclear pore complex that together with importin 7 (*IPO7*) activates the so called alternative pathway of hTERT entry to the nucleus (75). Interestingly, *IPO7* strongly influences the TERT branch also in LS-CRC, suggesting that in LS-CRC and MSI s-CRC, nuclear import of hTERT occurs via the alternative pathway (Supplementary Figures 12, 13) (53, 75).

These results, altogether, show that the TEL pathway is mainly activated through the TERT and DKC1 branches, by overexpression of *DKC1* and/or *TERT* genes in all CRC subtypes. Importantly, expression of *DKC1* is more prominent in MSS, than in MSI.

### Activation of ALT-TMM

Expression of the majority of genes of the ALT-TMM increases in all cancers studied compared with the reference mucosa with a large overlap between them (Figures 5, 6). On mean PSF-level, we found that the ALT pathway is activated in MSI s-CRC and partly also in G1 LS-CRC, but not in MSS s-CRC, and is paralleled by a markedly increased variability of the PSF-values of the ALT-branch compared with that of reference mucosa (Figure 4). In all cancer types, we noted activation of the HR branch of ALT-TMM, especially of step 2 and 3, and also of the APB branch compared to reference with larger amplitude in MSI compared with MSS s-CRC (Figure 3).

Activation of ALT in MSI s-CRC is mainly due to overexpression of *RAD51* (HR Step 1), *POLD3*, and *RFC1* (polymerase δ subunit, HR Step 2) which suggests activation of template directed synthesis of telomeres via the *RFC1-PCNA-POLD3* axis (41) (Figure 6). In addition, the APB branch component *PML* is overexpressed in MSI s-CRC. MSS s-CRC shows also specific up-regulation of a series of other APB-genes (*MRE11A, RAD50, SMC6*, and *NSMCE2*), and down-regulation of *SP100*, which has an inhibitory effect on ALT through sequestration of the MRN complex (*NBN, RAD50, MRE11A*) from APBs (Figure 6) (79, 80). Interestingly, *SP100* is found to be the only gene significantly down-regulated in G1 LS-CRC (Figure 6, Supplementary Figures 8A), which suggests a common function in G1 LS- and MSS s-CRC. Notably, SP100 differential gene expression between CRC tumors and reference mucosa changes in concert with transcriptional signatures of inflammation which indicates especially a marked decay in G1 LS-CRC due to immune escape driven tumorigenesis (45). *SP100* and *PML* accomplish also extra-telomeric functions related to inflammation and immune response (81), and oxidative stress reduction (82), which presumably overlay, or even couple with their roles in TMM (83). High immune cells infiltration is a characteristics of MSI s-CRC (84).

Generally, the top PI-values of the ALT-genes are markedly smaller (range −0.05–0.05) than that of the TEL-TMM (−0.2–0.2). This difference indicates an overall smaller influence of single genes on the ALT-TMM in units of PSF. In addition, we have observed stronger inhibitory effects (PI <0) of repressor genes in ALT, compared to TEL TMM (Figure 7). Among them, the chromatin modifiers *SUV39H1* and *SUV420H2* affecting chromatin decompaction (85), *ATRX* repressing ALT via the TERC/TERRA-instability branch (11), and Holiday junction resolvases *EME1* and *SLX4* that suppress telomere synthesis during ALT (19). Among the top activators of ALT-TMM are the nuclear receptor *NR2F2* and *ZNF827*, with NR2F2 promoting ZNF827-directed recruitment of the NuRD complex to telomeres (86); *BRCA1* and *PML*, genes involved in APB formation (87); and *POLD3*, encoding the catalytic subunit of DNA polymerase δ, involved in template directed telomere synthesis during ALT (41).

Hence, ALT seems to be affected by numerous genes, especially in MSI s-CRC, which concertedly adjust the activity of this pathway by activating and inhibitory influences of relatively small amplitudes. This is in line with the notion that the regulation of ALT is more complex and involves multiple layers of processes such as epigenetic modifications and homologous recombination events (12, 88), while TEL may be regulated in a simpler way by single factors, such as induction of *TERT* or *TERC* expression. Altogether, our data indicate that TEL is the major TMM in the CRC cases studied, while the ALT pathway additionally activates mainly in MSI s-CRC due to the concerted action of a number of factors, among them the HR and APB TMM branches as the main drivers.

### Gene Regulatory Networks in MSI and MSS s-CRC

**Figure 8:**
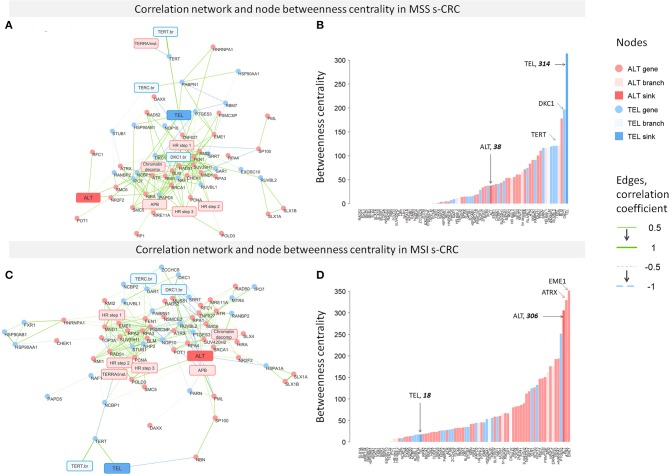
Correlation networks of TMM gene expression and ranked node betweenness centrality (BC) in MSS **(A,B)** and MSI **(C,D)** s-CRC. The edges of the network refer to Pearson correlation coefficients between gene expression and/or PSF-scores of the TMM nodes. It shows that the MSS network is dominated by a large number of connections linked to the TEL-node, while the MSI network is characterized by interconnections between ALT-nodes.

To assess the degree of co-regulation between the TMM-genes, we constructed pairwise correlation networks of expression values separately for MSI and MSS s-CRC (but not for LS-CRC because of small sample size). We included also the PSF-scores of the major sink nodes of the TEL- and ALT-branches of the TMM-pathways to directly evaluate correlations between branch and gene activities (Figure 8). The degree of interconnectivity of the nodes of the networks was then compared between the two s-CRC types using betweenness centrality (BC) as a measure (Figure 8). The distributions of BC-values of both s-CRC types indicate scale-free properties of the networks, which are characterized by a few highly interconnected “hub”-genes and/or -nodes accounting for most of the regulatory interactions and a large number of weakly connected genes/nodes.

More detailed inspection revealed pronounced differences between MSS and MSI s-CRC: most of the genes having high BC values in MSS s-CRC belong to the TEL pathway (e.g., *TERT* and *DKC1*), including also the TEL-sink node while the tail of the distribution showing low BC values accumulates ALT genes (Figure 8B). The reverse picture with highly connected ALT- and weakly connected TEL-genes and nodes is found for MSI (Figure 8D). In this subtype, the ALT-genes *EME1* (step 3 of HR branch) and *ATRX* (TERRA/Telomere instability branch) are strongest hub regulators according to their large BC values. This result is in line with the known fact that the chromatin re-modeler *ATRX*, being responsible for proper histone deposition at telomeres, acts as a key regulator suppressing ALT in many cancers, where however its deactivating mutation (as, e.g., in astrocytic gliomas) is not mandatory by unknown reasons. However, also a few TEL genes predominantly from the TERT- (*PTGES3, TERT, HSP90AA1*, see Table 1) and TERC- (*NAF1, PARN*) branches are obviously strongly involved into the network of this subtype suggesting coupling with ALT-TMM. Note also that *DKC1*, which is one of the strongest regulators in MSS s-CRC, nearly completely lacks interconnections in the MSI network, which is in line with the decreased activity of this gene in this subtype. Overall, these results suggest that the TEL pathway is more prone for activation in MSS, while ALT in MSI s-CRC according to the “guilt by association” paradigm assuming that co-regulated genes are likely to be involved in the activation of a biological process (89).

We do not observe separate clustering of TEL and ALT pathway genes in either of the subtypes, but rather a common network with a high degree of cross-connectivity suggesting mutually linked co-regulation of the two TMM processes. Interestingly, a number of anti-correlated and thus mutually repressive interactions were detected between TEL- and ALT-networks, especially in MSI s-CRC, e.g., between *ATRX* (TERRA branch) and *PTGES3* (TERT branch) both showing also highest BC-values which makes them candidates of regulatory links between TEL- and ALT-TMM. In summary, co-regulatory network analysis supports the notion of a more pronounced activation of TEL in MSS, and of ALT in MSI s-CRC (Figure 4), at the same time showing no clear-cut decoupling between the two telomere maintenance processes, but rather their coexistence, and co-regulation.

## Discussion

**Figure 9:**
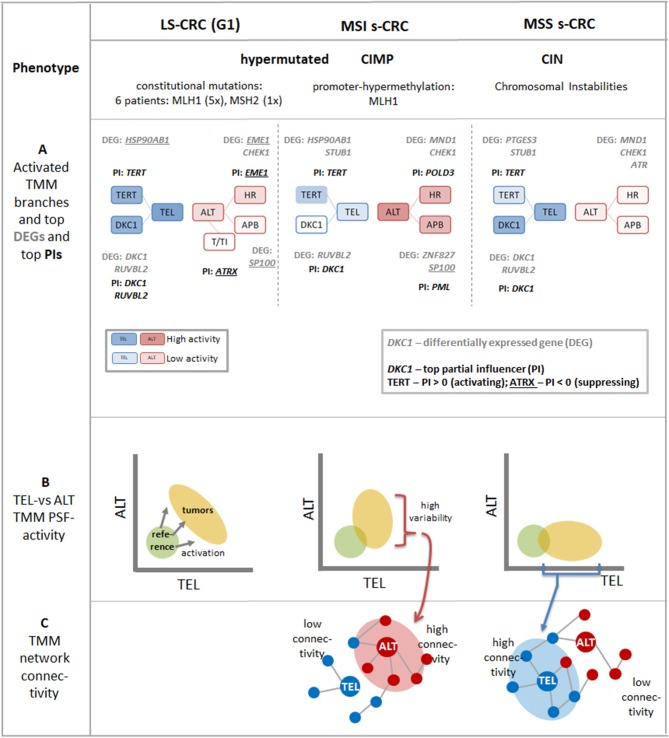
Schematic summary of the major aspects of TMM pathways in LS-CRC and in MSI and MSS s-CRC associating with different tumor phenotypes: **(A)** The transcriptional activation patterns of the TMM pathways lead to a shift from more active ALT-TMM in MSI s-CRC toward more active TEL-TMM in MSS s-CRC and concerted activation of both TMM in LS-CRC (see also Figure 3). The top differentially regulated genes (DEGs) and top partial influencers (PI) in cancer vs. reference tissues are depicted. The activity of the final TEL node is strongly “influenced” by *TERT* and *DKC1* genes while ALT is under control of a series of genes exerting activating as well as inhibiting effects of small and moderate amplitudes (see also Figure 7). Overall, *TERT* and *DKC1* are key factors leading to activation of TEL-TMM in all cancer subtypes studied while ALT TMM is affected first of all by APB, HR, and other subbranches of the TMM pathways. **(B)** The dark yellow ellipses schematically illustrate the distribution of tumor data. Their more distant position from the coordinate origin compared with the location of mucosa reference samples (green circles) reflects activation of TMM in the tumors (see also Figure 4). The decreased variations along the TEL and ALT axes reflect repression of these TMMs in MSI and MSS s-CRC, respectively. **(C)** Stronger activation of TEL- or ALT-TMM accompanies with markedly increased interconnectivities of the correlation networks formed between the genes and sink-nodes of these TMMs, respectively (see also Figure 8).

We have performed a combined study of telomere length and its transcriptional regulation in selected subtypes of CRC using bioinformatics analysis based on DNA and RNA sequencing data and using a TMM-pathway model. Our analysis provides insights into telomere length regulation in MMR deficient CRCs caused either by constitutional mutations mainly of the MLH1-gene in LS-CRC or by hypermethylation of the MLH1-promotor in MSI s-CRC, both leading to hypermutated cancer phenotypes (Figure 9). For comparison, we included MSS s-CRC cases forming a chromosomal instability (CIN) phenotype and specimen of non-tumor mucosa.

### Alterations of Telomere Length Indicate Tumor Onset but Are Virtually Insensitive for CRC Subtypes

We have found that all CRC-types studied had on average shorter telomeres than non-tumor colonic mucosa tissues, in agreement with previous reports (30, 32, 34, 61). Gene set analysis of transcriptomic data shows that accelerated cell division rates inversely relate to MTL until telomere length reaches a critical lower limit, which is then maintained after activation of TMM. This scenario is in agreement with the classical model of telomere maintenance. Accordingly, intensive proliferation of cancer cells leads to loss of telomeric caps, which triggers telomere crisis, and chromosomal instability and then drives early carcinogenesis (90–92) enabling cancer cells to bypass telomere-induced apoptosis by activating TMM just on a level which maintains the minimum critical telomere length required for survival (93).

Our observation that the transcriptional level of cell cycle-related genes is proportional to the activity of TMM genes also in non-tumor mucosa suggests that TMM becomes continuously activated in pre-neoplastic mucosa. This view is supported by the continuously decreasing distribution of MTL-values in reference mucosa without clear-cut separation with respect to MTL in the tumors. Moreover, all LS-adenomas show MTL near the minimum values observed in the LS-cancers. Overall these results support the view that telomere attrition is an early event in CRC tumorigenesis (94) and that early carcinomas arise from cells with critically short telomeres (95).

We find that the difference between reference tissue and tumor telomere lengths is larger in MSI s-CRC and LS-CRC, compared to MSS s-CRC which can be rationalized by higher telomere shortening rates in hypermutated tumors (31, 96), or, alternatively, also by earlier diagnosis and the younger mean age of LS- and MSI s-CRC patients, possessing on average longer telomeres in their reference tissues. We find slightly shorter mean MTL in MSI compared with MSS tumors, in agreement with (96), however at low significance level (*p* = 0.19), presumably due to our small sample size. Experiments on mice have indicated that dysfunctional TEL-TMM and MMR-defects can abolish anticancer activity of short telomeres via cell cycle related mechanisms (97).

### Telomeric Repeat Variants—Suited Markers for TMM?

Non-canonical telomere repeat variants (TRV) were found to cover up to 2% of the overall telomere length in the tumors and reference tissues studied in agreement with data on other cancer types (66). The most abundant TRVs detected are the substitution variant TGAGGG, previously reported in other studies (66, 98), and a novel insertion variant TTAGGGG. The slight increase of the relative amount of TRVs in tumors (by up to 1.5%) can be rationalized by biased accumulation of TRVs in the proximal telomeric regions virtually not affected by telomere attrition (98).

Only a few studies have explored the difference between TRV generation in tumors with activated telomerase or ALT so far (63, 66), to the best of our knowledge. They have reported differences in TRV abundances between TEL and ALT TMM, mostly based on cell line systems. TEL, on one hand, is found to induce substitutions at repeat positions 1 and 3 due to improper telomerase function (63). ALT, on the other hand, seems to induce random placements of TRV arising from proximal and terminal regions of telomeres via homologous recombination (63). Later, the same group has classified ALT positive(+) from ALT negative(−) cell lines, based on relative TRV content and relative telomere length (66). Most of the ALT-related TRVs had lower relative TRV content, largely attributed to longer telomeres in these cell lines and to “proximity effect.” We found a similar trend in MSI-vs.-MSS comparisons (Supplementary Figure 6) which corresponds to the slightly enhanced ALT-TMM expression signature in MSI s-CRC reported by us. Interestingly, all the TRVs, except for TTCGGG behaved similarly, showing reduced relative content in MSI vs. MSS s-CRC, in agreement with ALT+ vs. ALT- differences observed in Lee et al. (66) (Supplementary Table 3B). Overall, our TRV analysis thus agrees with the previous reports regarding the basic trends to distinguish ALT-vs.-TEL TMM in agreement with our transcriptomic data.

Importantly, TRV studies based on sequencing data are still (very) rare. Absolute quantification of TRV lengths requires systematic methodical studies. Computational telomere and especially TRV length estimates should be interpreted as subjective measures with possible off-sets between the methods. The different approaches in these methods, such as telomeric read capture [alignment (25) vs. repeat count with differing count thresholds (63, 66)] may lead to capturing subtelomeric and interstitial telomeric repeats at varying degrees, which may eventually affect absolute TRV length and relative content. Consequently, they provide consistent quantitative results only within each method used. TRV-estimates are expected to be prone to systematic shifts due to varying GC-content and G-stack formation with strong effects on hybridization chemistry (99) and possible consequences for read-count estimates.

Overall, our results and previously reported findings underline the need for further studies on association of TRV composition with TMM activation across cancers in general and in CRC subtypes in particular. Moreover, the small amplitude of TRV changes and confounding factors affecting, e.g., age and telomere length and their overlay with TRV-proximity effects leaves a series of questions still unanswered.

### Different Levels of Expression Analysis Provide Consistent TMM-Related Transcription Patterns Specific to CRC Subtypes

Gene expression data were analyzed making use of pathway models considering a set of 67 genes with relevance for TEL- and ALT-TMM. Analyses have been performed at four levels addressing different aspects of transcriptomic regulation (Figures 9A–C): (i) Differential expression analysis, as the most “simple” approach, was applied to estimate expression differences of the genes between cancer and reference mucosa and between the cancer subtypes, as independent entities; (ii) Pathway signal flow (PSF) analysis, has been used to estimate the activity of genes in a certain pathway topology considering their mutual interactions; (iii) The partial influence (PI) was applied to estimate the specific impact of a selected gene on a certain node of the pathway; (iv) Finally, correlation network analysis enabled us to select co-expressed and thus potentially co-regulated genes in an unsupervised fashion, i.e., without assuming a predefined wiring between them.

In all these analyses, we separately considered the TEL- and ALT-TMM in order to compare their particular impact on each of the CRC subtypes. For this purpose, we generated biplots of their pathway activities (Figure 9B) and provided TEL-and ALT-specific lists of top genes in units of differential expression, PSF, PI, and BC, respectively (Figures 9A–C and Table 1). This parallel view on both mechanisms was motivated by recent research indicating that categorization of tumors into either TEL- or ALT-positive ones appears to be imprecise. In other words, tumors do not necessarily classify into exclusively a single TMM-type. Particularly, TEL- and ALT- TMM can coexist either in different cancer cell sub-populations of the same tumor (12) or within the same cell (15). Moreover, TEL- and ALT-TMM are capable of switching from one mechanism to the other one during different stages of tumor development or upon treatment (15).

Our results support this view. We find concordant activation of both TMM pathways in all the CRC subtypes studied compared with the reference mucosa systems, showing no clear-cut separation between samples in terms of either TEL or ALT pathway activation (Figure 9). TEL seems to be the dominating TMM in all analyzed CRC subtypes. However, the branches leading to activation of hTERT (TERT branch) and dyskerin (DKC1 branch) contribute differently with distinctly stronger mean contribution of DKC1 in MSS compared with MSI s-CRC. In turn, ALT-TMM shows stronger effects in MSI compared to MSS s-CRC; mainly via APB formation (APB branch) and homologous recombination events (HR branch) (Figures 9A,B). Regulation of ALT pathway is more complex than TEL and involves multiple events. Strikingly, the two TMMs show strong co-regulation of member genes.

Notably, higher mean activity of ALT-TMM in MSI CRCs is accompanied by higher variability of the ALT-PSF values in these samples *and* stronger co-regulations between the ALT-genes in the gene network. Such co-regulations are indicated by higher network connectivity in these CRCs compared with MSS s-CRC, where the relations between these characteristics are reversed. These results stand for a possible trend of increased sensitivity for ALT in MSI and of TEL in MSS s-CRC, which, in turn, can reflect repressive feedback mechanisms between TEL- and ALT-TMM presumably mediated by anti-correlated links detected in network analysis especially in MSI s-CRC. On the other hand, co-activation of TEL and ALT in the tumors, strong co-regulation between the TEL- and ALT-TMM genes and positive correlation of both TMM with cell cycle activity and other cellular processes, indicate that mutual activation of TEL and ALT-TMM is possible in most of the cancer samples. All together, these results support the notion of a TEL-ALT continuum of expression and pathway activation patterns, where both pathways are concertedly regulated in a fine interplay of activating or mutually repressive interactions. This kind of regulation eventually leads to a situation, where TEL and ALT can co-exist in the same tumor, although at different activity levels. These levels can be specific for each tumor subtype.

### TMM Genes as Markers of Telomere Attrition and Limitations of the Study

LS-CRC (G1) and MSI s-CRC reveal an increased mutational load compared with MSS s-CRC including the TMM genes (45). However, only few of them were mutated on moderate recurrence levels of <50% mainly in the APB branch of ALT-TMM (Supplementary Figure 10). Hence, mutation markers seem not to be suited for judging tumor development, subtypes and/or TMM in CRC. This contrasts to other cancer types, such as gliomas that show strong association between astrocytic and oligodendroglial subtypes and telomere biology, which is driven mainly by mutations of the *ATRX* and *TERT* genes, respectively (100), as well as aggressive metastatic melanomas (101) and other cancers [see (102) and references cited therein] showing a high percentage of *TERT* mutations.

According to our results, RNA-seq data has the promise to offer an alternative and independent option for judging the telomere status of CRC. Fortunately, they are available in many molecular cancer studies. Frequently, *TERT* is used as a gene expression measure of TMM activity, e.g., to estimate tumor progression in CRC [see (28–30, 30–33) and references cited therein]. Here, we found significant differential expression of *TERT* between s-CRC tumors and reference. However, TERT showed by far not the largest effect (position 23, 29, and 31 in the ranked lists of 67 DEGs in MSS, MSI and LS, respectively; see Supplementary Table 5). Because of multiple extra-telomeric functions of *TERT*, by *TERT*-bypassing mechanisms of tumor development (103) and because of subtle epigenetic regulatory mechanisms of *TERT* activity (104). Moreover, whether TERT expression translates directly to telomerase activity is unclear because only the full-length transcript (as opposed to known isoforms) has been found to activate telomerase (105, 106). Thus, the transcriptional level of this gene may not serve as a stable indicator of TEL pathway activity. We found that other transcripts, such as *RUVBL2* (telomerase complex assembly), *DKC1* (telomerase subunit) and also *HSP90AB1* (*TERT* nuclear import), show much stronger and more consistent effects in our TEL-TMM data making them suited candidates for estimating TEL-activity (Table 1). Interestingly, *DKC1* (and partly also *RUVBL2*) overexpression associates consistently with unfavorable prognosis in renal, liver, head-neck, endometrial and skin (melanoma) cancers (107, 108). We expect these transcripts to function as potential markers with prognostic impact also in CRC.

The partial influence (PI) of *TERT* on TEL pathway activity is highest in all cancer subtypes in contrast to *TERT* differential expression, presumably due to the stabilizing effect of the interaction partners of *TERT* in its local pathway topology. *TERT* also occupies top positions in the betweenness centrality rankings. These two measures together show that consideration of pathway topology and/or degree of co-regulation will increase the impact of *TERT* as TEL-TMM marker. Please, recall also that MTL levels off at shortest boundary values in cancers, which makes it virtually insensitive to cancer progression, while expression of many TEL-genes is still considerably variable, thus making them potentially more sensitive markers for cancer development (Supplementary Figure 11).

Limitations of our study are linked to the relative small sample size, which decreases resolution especially of the MTL and TRV data obtained from whole genome DNA sequencing. On the other hand, our dataset of matched tumor-reference and combined whole genome DNA-seq and RNS-seq of LS-CRC is the only presently available data of its kind, to our best knowledge. So it represents a unique data source of this relatively rare disease (about 3% of bowel cancers). It is well-characterized in terms of subtypes, somatic and constitutional mutations and transcriptional states (45) and, it is reviewed as state of the art study addressing molecular heterogeneity of LS-CRC and providing novel insights into immune escape mechanisms of carcigogenesis of LS-CRC (46). The latter review emphasizes the need for identification of suitable molecular markers for describing tumor development and heter6ogeneity in these cancer types (46). The present study, despite its relative small size, provides a potential starting point for the search of such markers with focus on telomere biology. Please note also, that sub-stratification into molecular subtypes is an intrinsic problem in molecular cancer studies because they naturally reduce sample size in the strata. On the other hand, G1 and G2 behave similarly concerning telomere lengths what, in turn, increases significance in a combined view on the data (Supplementary Table 4A).

The supervised pathway approach restricts our results to a limited number of TMM genes selected and curated based on literature knowledge. Our conclusions regarding TEL/ALT-TMM activation thus refer to expression data and the pathway model applied. In a general sense they are not definite, but are indications of trends that have to be further validation by independent experimental approaches. Because of pleiotropic roles of many of these genes, e.g., related to extra-telomeric cellular functions accompanying telomere shortening, their particular function for TMM remains ambiguous in many cases and requires further studies. Selection, specification and extension of genes considered and adjustment of their interactions in terms of pathway topologies, together with systematic study of other cancer entities, are expected to improve the functional understanding of TMM and its impact in the context of tumor biology. Overall these analyses illustrate the general problem, namely that there is no clear-cut separation between “telomere biology” and other cellular functions. Pathways in general (i.e., not only our TMM pathways), represent models which consider direct interactions between genes and proteins on one hand but on the other hand focus on a definite “cutout” of cellular function which neglects relations to functionalities outside this “window.” This is their strength on one hand, but also their weakness. Such pathway models have been proven in many applications because of their focused view, which allows description of selected biological processes by means of definite ingredients. Our approach is only a first step in this direction, which needs improvement in future work. On the other hand, application of TMM-pathway models to “real world” data such as CRC omics data as done here are needed for such improvements.

## Conclusions

The present study demonstrated that genome and transcriptome sequencing can provide a detailed picture of alterations of telomere length, sequence composition and of gene expression changes related to transcriptional regulation of telomere maintenance in selected CRC subtypes. Thereby, gene expression data can provide an alternative to genomic data and/or complementary measure of the telomere status in tumors. Consideration of interaction topologies in pathway analysis provided additional information about the mechanisms of telomere length regulation in addition to standard gene expression analysis. Our study thus provides an example how omics data can support understanding of selected aspects of tumor biology.

## Data Availability Statement

The datasets analyzed for this study can be found in the dbGaP database (www.ncbi.nlm. nih.gov/gap) under accession number phs001407.

## Author Contributions

LN and HB conceived this study, performed analyses, and wrote the paper. LH performed mutation and gene set analysis. All authors contributed to data generation, methods development, and read and approved the final version of the manuscript.

### Conflict of Interest

The authors declare that the research was conducted in the absence of any commercial or financial relationships that could be construed as a potential conflict of interest.
