# Supplementary material for: Telomere Length Maintenance and Its Transcriptional Regulation in Lynch Syndrome and Sporadic Colorectal Carcinoma
Source: Front Oncol. 2019 Nov 5;9:1172. doi: 10.3389/fonc.2019.01172 (PMC6848383; doi:10.3389/fonc.2019.01172)
Supplement: Supplementary file 1 [file Data_Sheet_1.pdf]

Supplementary Material

Telomere length maintenance and its transcriptional regulation in Lynch syndrome and sporadic colorectal carcinoma

Lilit Nersisyan [1*], Lydia Hopp[2], Henry Loeffler-Wirth[2], Jörg Galle[2], Markus Loeffler[2,3], Arsen Arakelyan[1], Hans Binder[2*]

[1] Group of Bioinformatics, Institute of Molecular Biology, National Academy of Sciences, Yerevan, Armenia

[2] Interdisciplinary Centre for Bioinformatics, Leipzig University, Leipzig, Germany

[3] Institute for Medical Informatics, Statistics and Epidemiology, Leipzig University, Leipzig, Germany

*** Correspondence:**
Lilit Nersisyan: l_nersisyan@mb.sci.am, Hans Binder: binder@izbi.uni-leipzig.de

Content

1 Supplementary Methods..3
1.1 TMM genes and TEL- and ALT-pathways 3
2 Supplementary Figures and supplementary results...4
2.1 CRC subtype characteristics and 'partial influence'...4
2.2 Age dependence of mean telomere lengths and repeat variant analyses 6
2.3 TMM pathways, differential expression and mutation analysis 11
2.4 Partial influences of TMM genes...16
3 Supplementary Tables...18
3.1 Supplementary Table 2. Genes included in TEL and ALT TMM pathways [a]......................18
3.1 Supplementary Table 2: Verification of TMM genes using gene sets of the gene ontology (GO) category Biological Process (BP) and Cellular Component (CC) 20
3.2 Supplementary Table 3: Verification of TMM genes using TelNet database information (8) [a] 22
3.3 Supplementary Table 4: Telomere and TRV length analysis 30
4 References 34

1 Supplementary Methods

1.1 TMM genes and TEL- and ALT-pathways

There are several options to study transcriptomic regulation of telomere maintenance. Standard case-versus-control (e.g. samples with longer versus shorter telomeres) differential analysis is not the optimum choice because of strong coupling between TMM and many cellular processes, which gives rise to confounding effects with non-telomeric functions. For example, the list of differential expressed genes cancer-versus-reference of LS-CRC (60 genes, see (1) overlaps with a list of 67 curated TMM genes (see below) only in one position, because LS-CRC genesis is mainly driven by an immune escape mechanism virtually without direct relation to telomere biology. Because of these confounding effects recent telomerase-activity expression signatures are highly enriched in genes of cell cycle, RNA-processing and embryonic stem cell functionalities suggesting only limited direct impact for telomere biology (2,3).

As an alternative option one can use curated gene sets with direct impact for telomere function, e.g. as provided by gene ontology categories (12 gene sets). These sets were selected solely by biological knowledge without considering the type of effect (e.g. activating or repressing) and without testing their suitability using expression data. Here we pursue a semi-supervised approach which is based on literature search for genes related to TMM, their processing in a pathway topology with activating and repressing interactions which is proven using expression data of cell line and cancer tissue systems with activated TEL and/or ALT TMM (see (4) and (5) for details). In short: in a first step, TMM-relevant genes were selected by literature search based reports on protein-protein and protein-RNA interactions that lead to activation of the ALT and TEL TMMs (Supplementary Table 1). Overall this search came up with 67 genes which were assembled into two separate pathway graphs for TEL- and ALT-TMM activation according to the interaction topology reported in the literature (Supplementary Figure 7). Second, publicly available microarray gene expression data of ALT positive and TEL positive cell lines (as annotated according to the presence of APBs/C-circles and telomeric-repeat amplification, respectively (4,5)) of different tissue origin (N= 10), and liposarcoma tumor samples (N= 17) along with human Mesenchymal Stem Cells of healthy individuals (N=4) were taken from the GEO-repository (GEO accession number: GSE14533 (6)). The expression data were then used for fine-optimization of the pathway topology using pathway signal flow values as criterion (see main text) and an iterative algorithm which provides a classification accuracy of 100% for the cell line and of 75% for the liposarcoma specimen.

For independent verification of the TMM-genes available from (4,5) we applied two approaches:

1) We calculated enrichment of gene sets of independent functional assignments provided by the GO categories CC and BP taken from (7). Overall, this analysis clearly indicates strong accumulation of genes with explicit relation to telomeric function on all levels of our TMM-pathway topologies (Supplementary Table 2).

2) TelNet database information (8) also confirms the TMM-gene functions and their relevance for telomere biology (Supplementary Table 3).

2 Supplementary Figures and supplementary results

2.1 CRC subtype characteristics and 'partial influence'

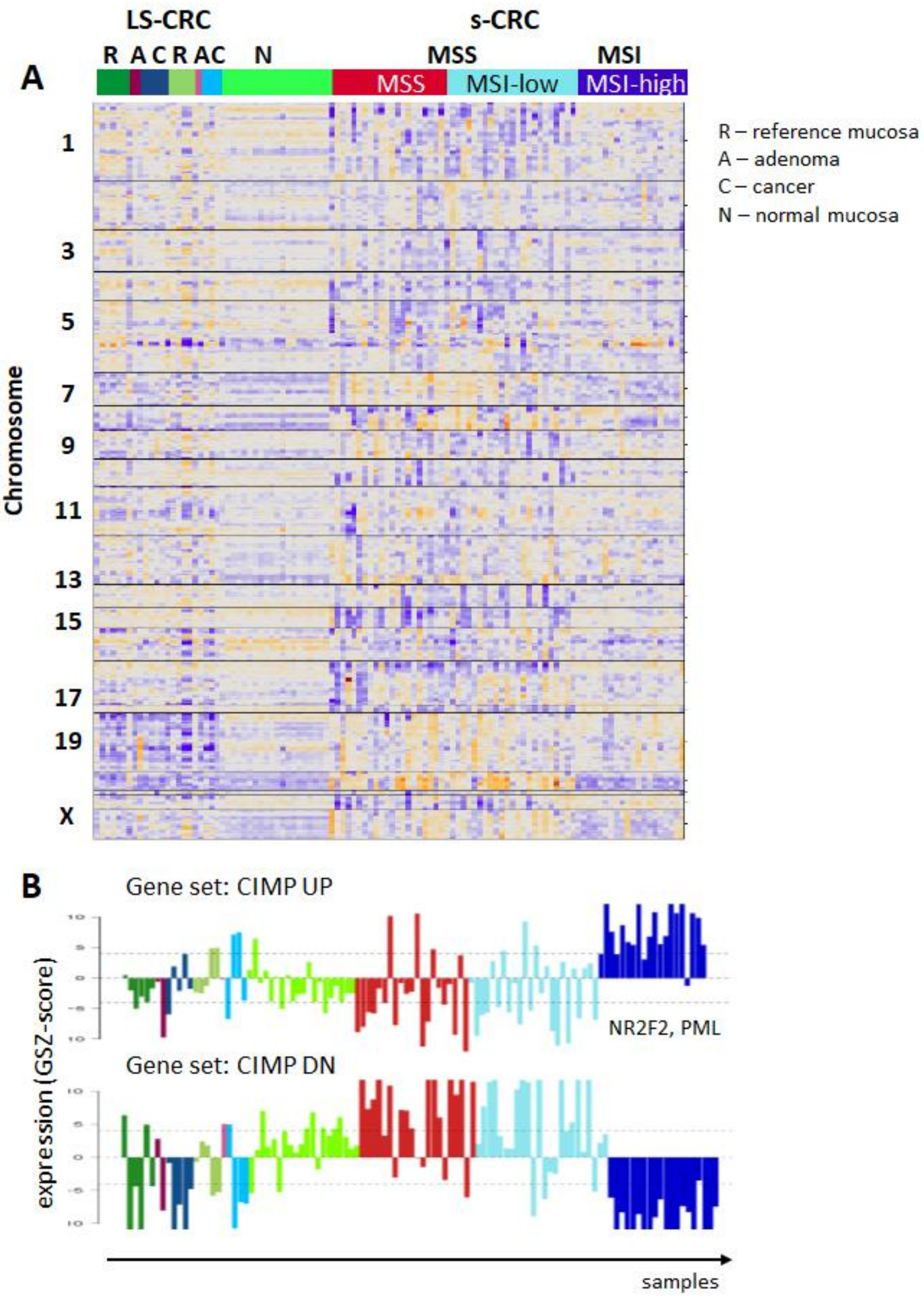

Supplementary Figure 1. Gene expression analysis assigns MSS s-CRC to a copy number instability phenotype (CIN) and MSI s-CRC to the CpG island hypermethylation phenotype (CIMP): A) Gene expression along the chromosomes was estimated using sliding-windows averaging to study positioning effects of the genes (9). Chromosomal gene expression commonly reveals a coarse pattern of up- (dark yellow) and down-(blue) regulated regions which indicate chromosomal regions of copy number gains and losses, respectively. Both, MSS- and MSI-low s-CRC subtypes can be assigned to the copy number instability phenotype (CIN), while the hypermutated MSI s-CRC subtype shows a smoother patterns reflecting less pronounced copy number instabilities. Note the common copy number loss at the short arm of Chr. 17 among the CIN-tumors. It harbors the *TP53* gene, a general tumor driver with loss of function in 50-75% of all CRC (10). Another CIN-feature is the copy number loss at Chr. 18 observed in 70% of primary CRC (10). B) The expression profiles of gene sets positively (CIMP UP) and negatively (CIMP DN) related to CIMP phenotype (11) assigns MSI s-CRC to the CIMP phenotype. MSS and MSI-low samples overall show similar expression levels thus supporting their collection into one MSS group. Note that genes PML and NR2F2, both being part of the ALT-TMM pathway, are part of the CIMP_UP signature, which gets up-regulated in CIMP s-CRC typically by DNA-hypomethylation of the gene promoters. We also analyzed a DNA-methylation data set of 22 LS-CRC cancer patients and 15 normal mucosa samples (GEO accession no. GSE 128607) and found no TMM gene among the about 2,000 differentially methylated (hyper- and hypo-methylated) genes (data not shown). The LS-CRC samples revealed marked CIMP characteristics similar to CIMP of s-CRC.

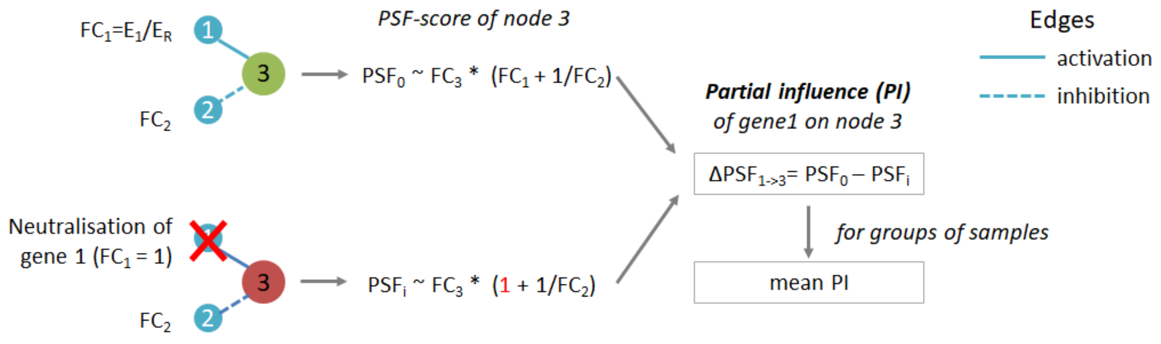

Supplementary Figure 2. Schematic sketch illustrating the partial influence (PI) of gene no.1 on gene no.3 in a simple network motif of three genes. The PI is defined as the difference of the PSF scores of gene no.3 before and after neutralization of differential expression of gene no. 1 by setting its FC_1=1. One sees that the sign of PI is positive/negative for activating/repressing effect of genes. For groups of samples the PI-values were averaged.

2.2 Age dependence of mean telomere lengths and repeat variant analyses

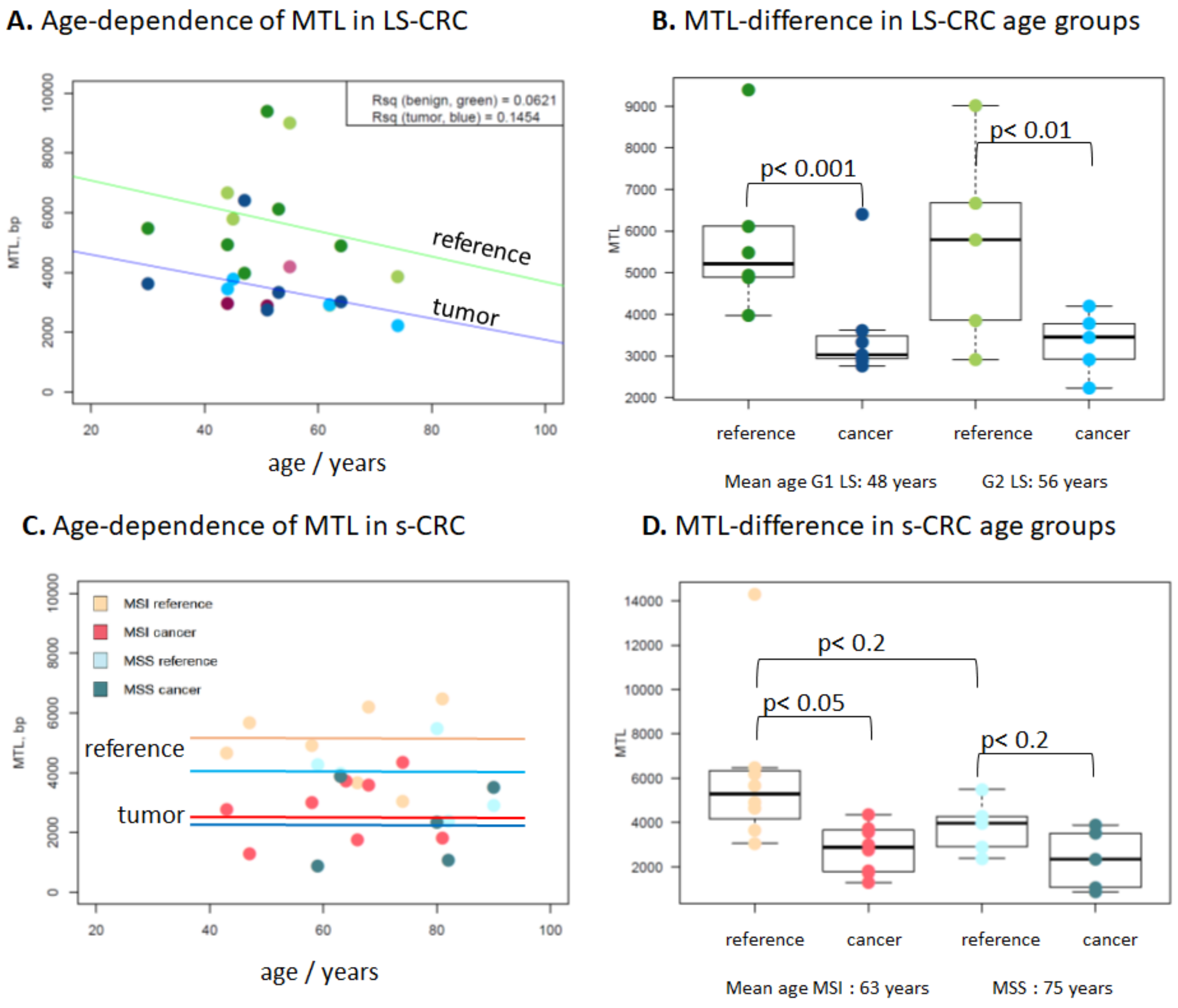

Supplementary Figure 3. Age dependence of mean telomere length (MTL) in LS- and s-CRC patients. The plots in the left part show the MTL of the specimen as a function of age. The MTL of LS-CRC patients decay with age as indicated by the virtually parallel regression lines for reference mucosa and cancer specimen (part A, see part B for assignment of colors to sample groups). For s-CRC virtually no decay could be detected by linear regression (C). The horizontal lines visualize the mean MTL in cancer compared with reference mucosa and for each of the systems studied. The boxplots in (D) reveal significant shortening of MTL in LS- and s-CRC compared with reference mucosa and also shorter telomeres in MSS compared with MSI s-CRC (paired Wilcoxson ranked test p's) which associates with elderly patients in MSS compared with MSI on the average. See also Supplementary Table 4A for MTL data.

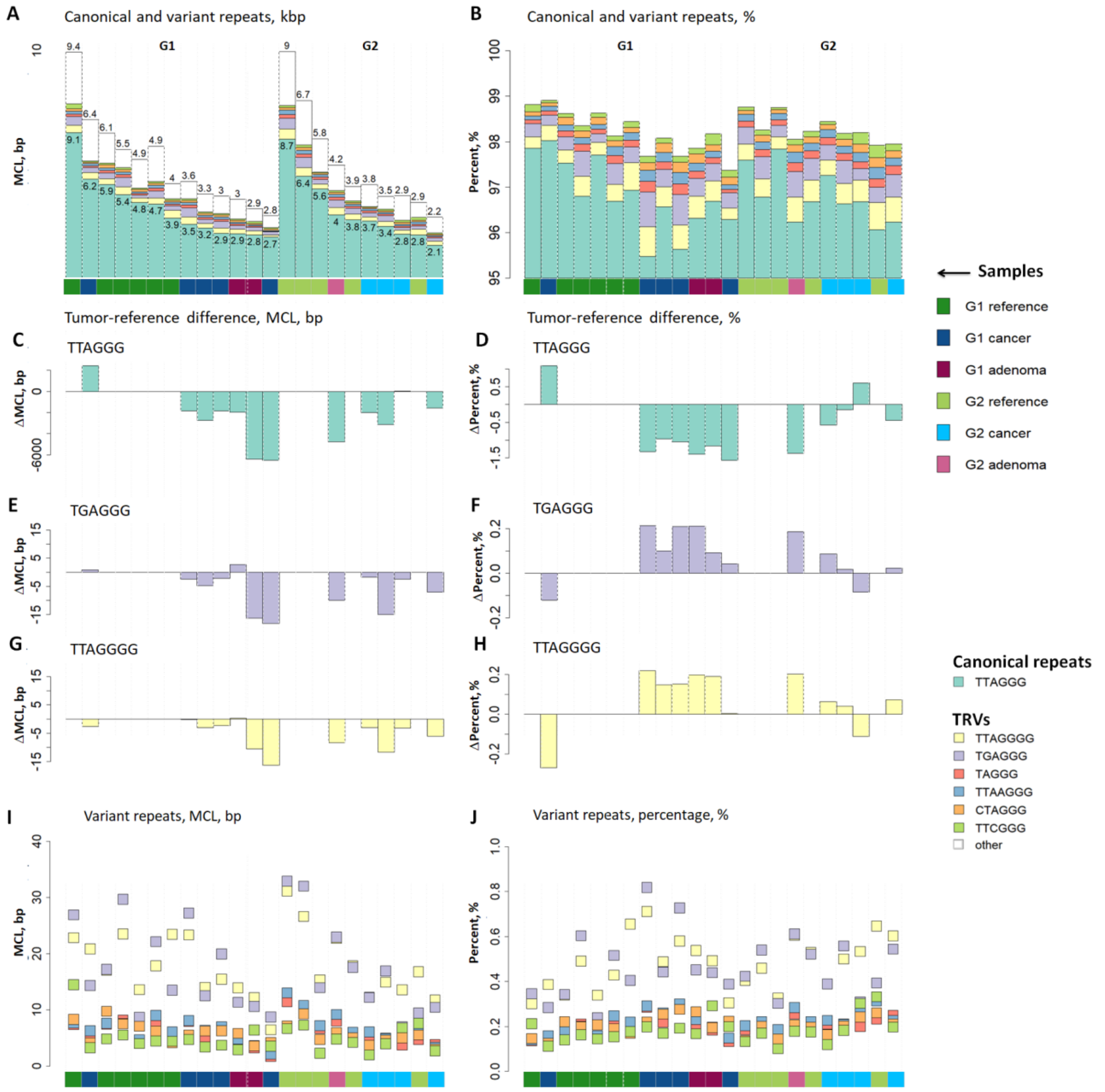

Supplementary Figure 4. Canonical telomeric repeats and telomeric repeat variants (TRVs) in LS CRC: Mean cumulative lengths per chromosome (MCL, left part, in units of bp) and the percentage (right part) of the canonical and the most abundant TRVs in G1 and G2 LS CRC. A: Stacked barplots of the composition where the total heights of bars correspond to MTL (see numbers in units of kbp), while the MCL of canonical variants is indicated with numbers underneath. B: Percentage of the canonical and the of the top six TRVs. C-H: tumor versus reference tissue difference in MCL and percentage of the canonical TTAGGG repeats (C-D) and the most abundant non-canonical variants TGAGGG (E-F) and TTAGGGG (G-H). One sees that MCL and the percentages of canonical variants to decrease while the percentages of TRVs predominantly increase which all together indicates the increasing amount of TRVs upon telomere attrition. I-J: MCL and percentage of the top six non-canonical variants. Most abundant TRVs are the G-insertion/substitution variant TTAGGGG and TGAGGG. Overall, the non-canonical variants are rare with less than 1% of MTL, and almost invariant MCL (less than 15 bp reduction on the average) in tumors.

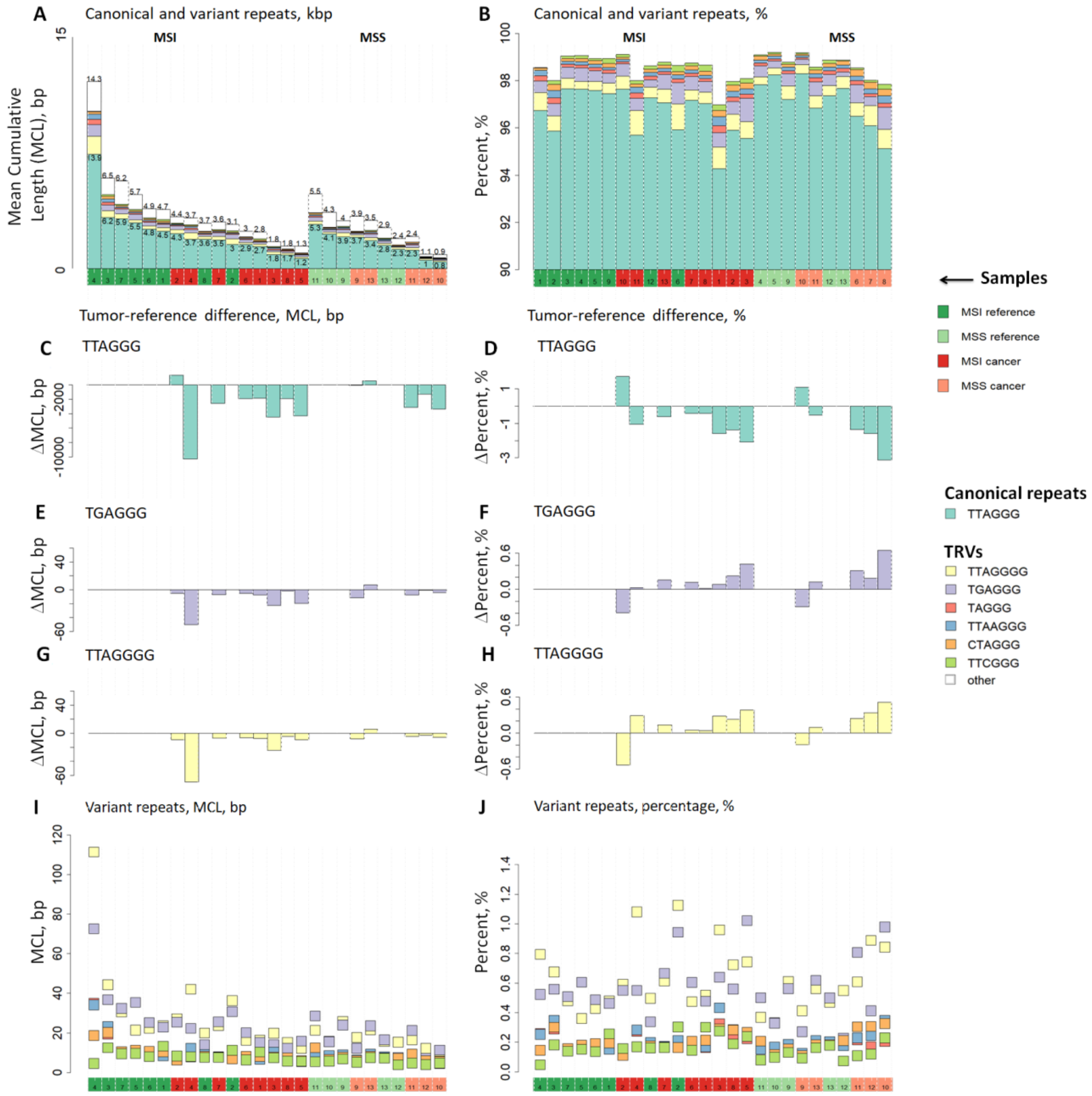

Supplementary Figure 5. Canonical telomeric repeats and telomeric repeat variants (TRVs) in MSI and MSS s-CRC. See legend of Supplementary Figure 4 for explanations. Overall the results for LS-CRC and s-CRC are very similar.

A. Differential length analysis of TRVs in s-CRC

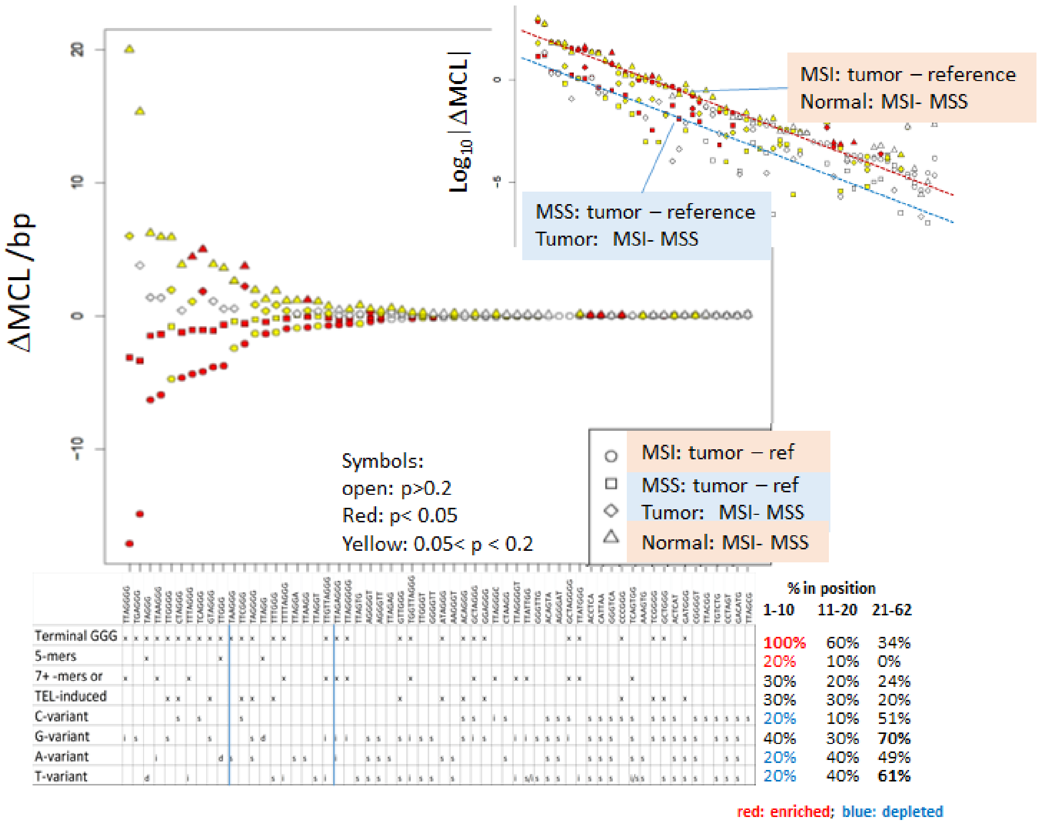

B. Absolute and relative TRV content in s-CRC

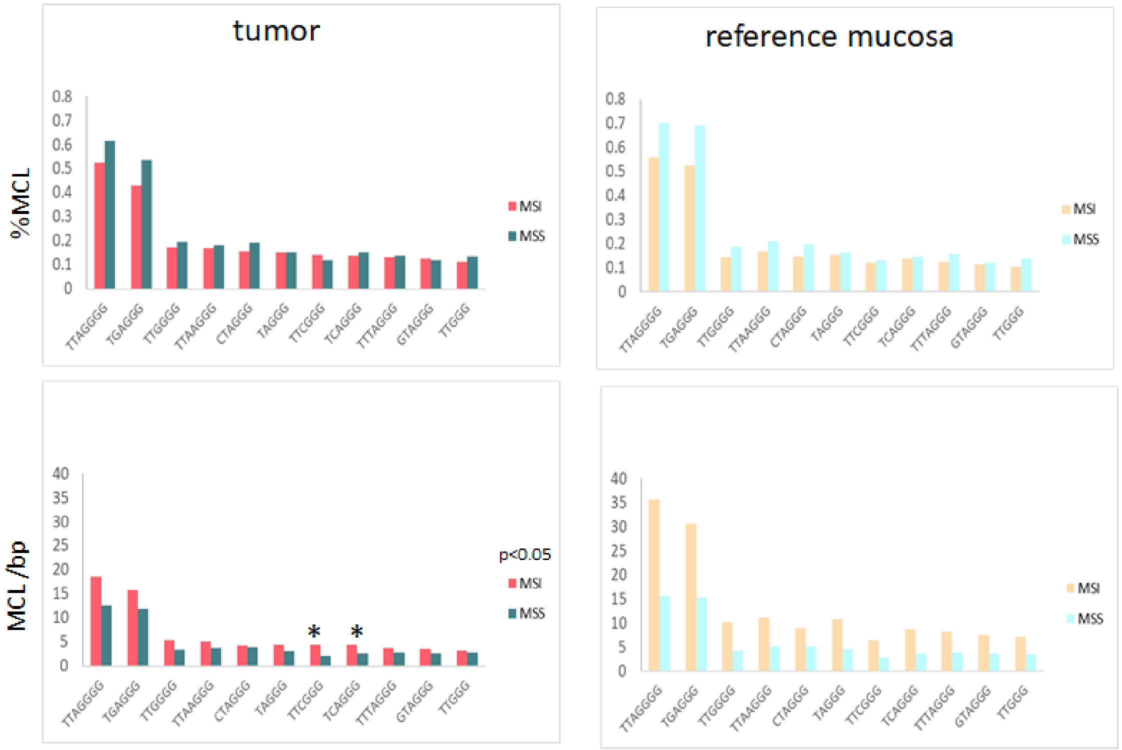

Supplementary Figure 6: Extended TRV-length analysis: (A) Differential mean cumulative TRV-lengths (ΔMCL) between tumor and reference and between MSI and MSS in s-CRC are ranked with decreasing ΔMCL of tumor – reference in MSI s-CRC. The ΔMCL values of the other differences all roughly follow this course indicating similar rankings of the TRVs in the different systems. Interestingly, the first about one dozen most abundant TRV contain the terminating 'GGG' motif, which supports the notion that this sequence is under strong selective pressure (12). Further, 5meric TRVs accumulate slightly among the top ones while A-, C- and T-variants deplete compared with their appearance at lower ΔMCL-levels. Variants, attributed to TEL-induction are virtually evenly distributed. The plot in logarithmic scale shown as insertion further revealed almost linear decays even for tiny ΔMCL values. The logarithmic decays can be roughly described by two mutually parallel lines. The upper one refers to the ΔMCL of tumor minus reference in MSI and to the difference between MSI and MSS reference mucosa while the lower one refers to ΔMCL of tumor minus reference in MSS and the difference between MSI and MSS tumor samples. (B) Absolute and relative lengths of the top TRVs in MSI and MSS s-CRC. Two TRV are significant different between MSI and MSS s-CRC (marked with asterisk, see also Supplementary Table 3B). The fact that we observe similar differences between MSI and MSS in reference mucosa reflects similar TRV composition in the reference system, which get modified mainly by proximity effect and telomere shortening in tumors (see Supplementary Figure 5) and which eventually suggest presence of differences in molecular mechanisms with impact for TEL and/or ALT almost in pre-malignant tissues.

2.3 TMM pathways, differential expression and mutation analysis

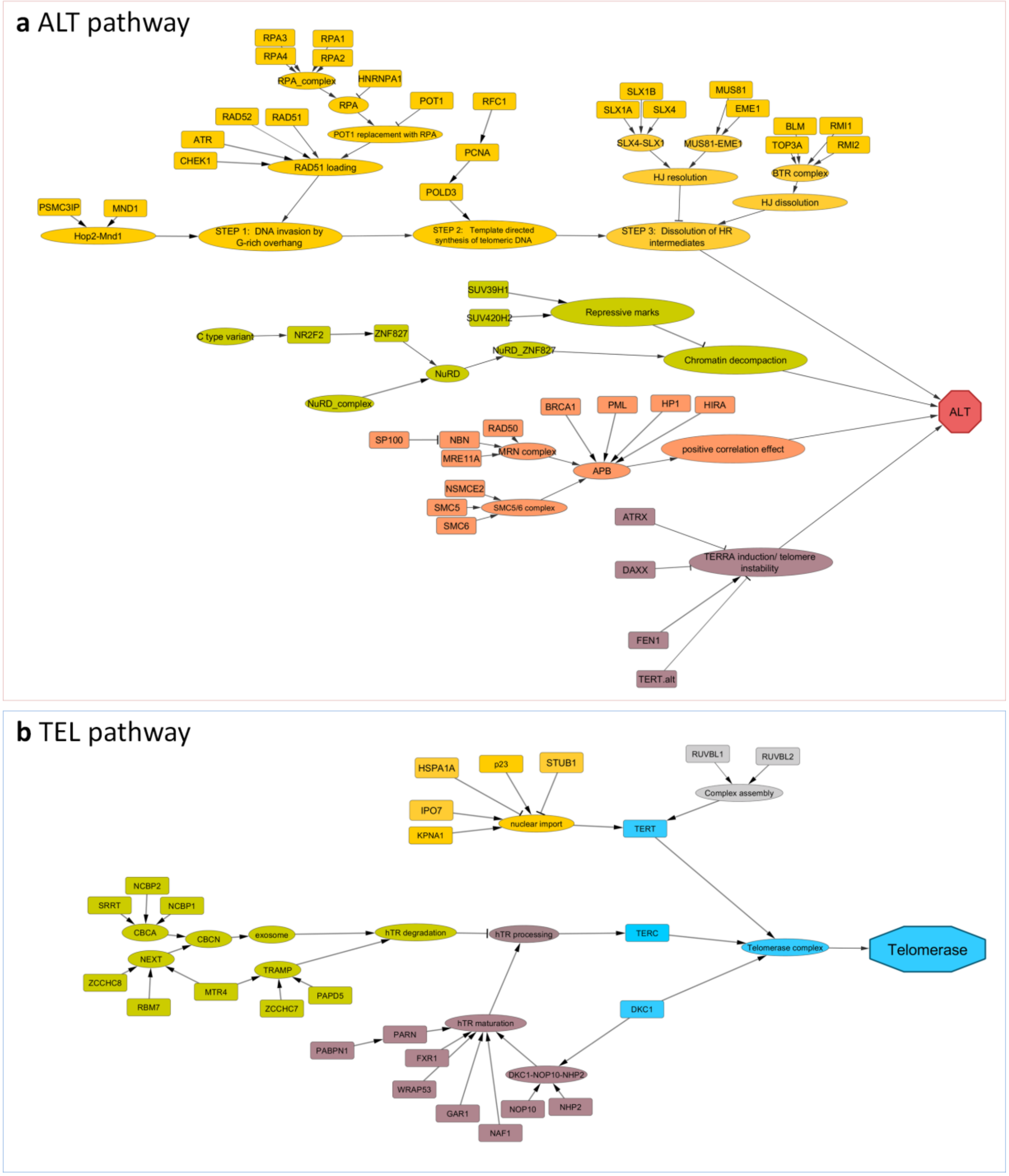

Supplementary Figure 7: The TEL (a) and ALT (b) pathways. The “Telomerase” and “ALT” nodes are the targets of each pathway. In (a), the core components of the telomerase complex are colored blue. The rest of the colors in both plots are for enhancement of readability. Rectangular nodes represent genes, while oval-shaped nodes represent processes or complexes. The edges with delta and T shaped targets indicate activation and inhibition interactions, respectively. For a detailed description see (4,5).

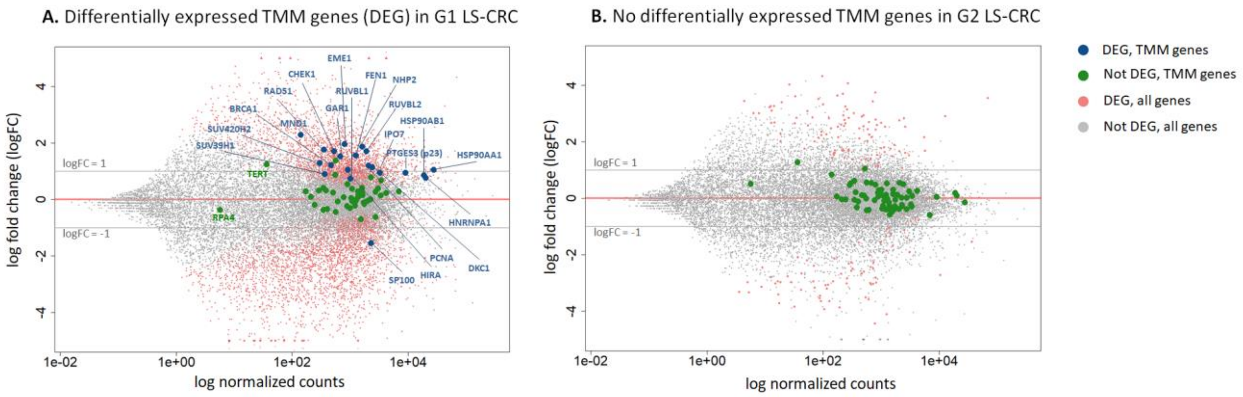

Supplementary Figure 8. Plots of differential gene expression (log2 fold changes of read counts) in G1 and G2 LS-CRC versus reference mucosa, as a function of gene expression (log-normalized read counts). The differentially expressed TMM genes (DEGs, adjusted $p < 0.05$) are colored blue, the rest of TMM genes are green. In G2 no gene meets the significance threshold (‘no DEGs’).

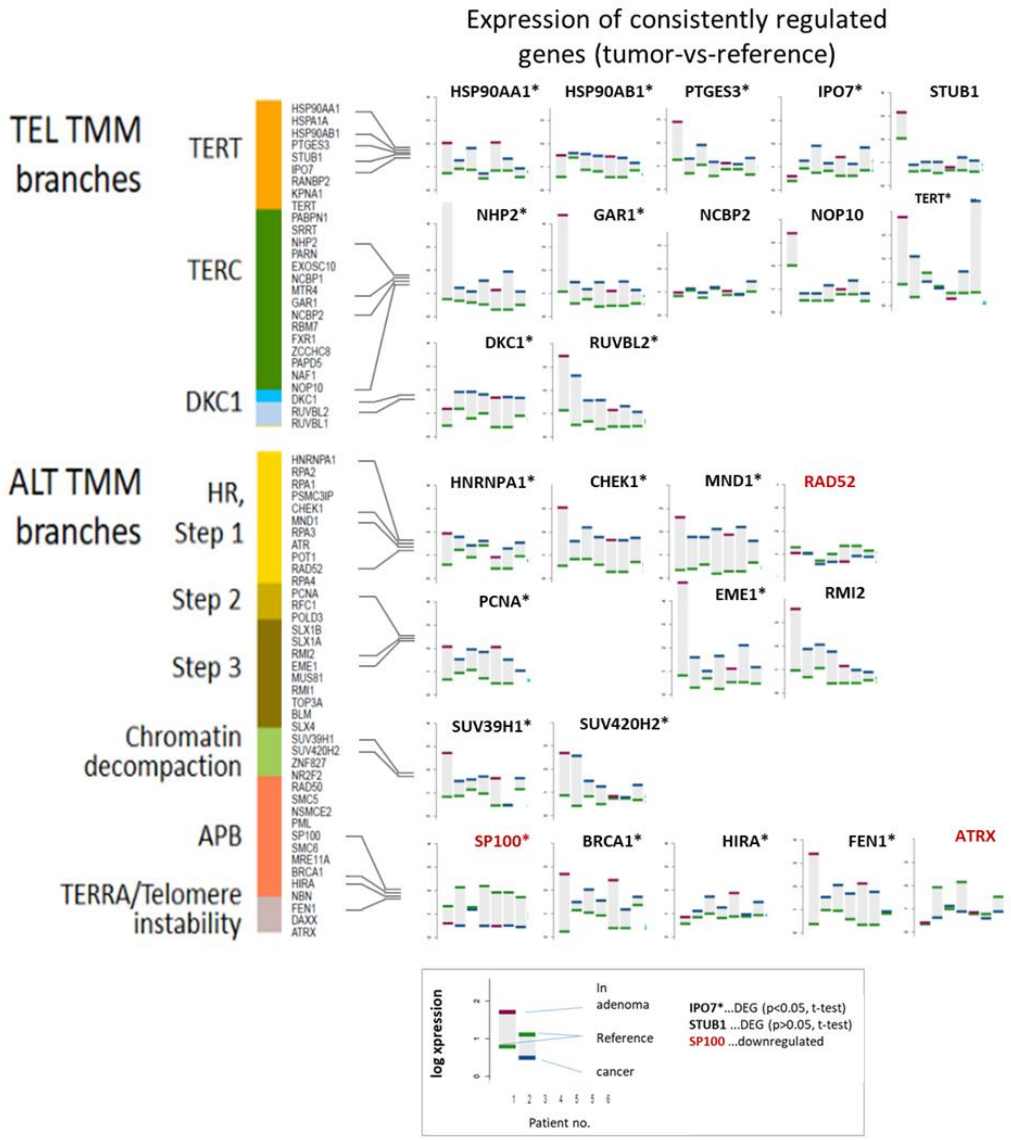

Supplementary Figure 9. Differential expression of selected genes in G1 LS-CRC with respect to patient-matched reference mucosa. The genes selected show mostly consistent changes of gene expression in most of the patients. Note the large variance of *TERT*-expression in contrast to, e.g. *DKC1*, *RUVBL2* (DKC1 branch of the TEL TMM pathway) and *CHEK1*, *MND1* and *PCNA* (HR branch of ALT-TMM) showing similar alterations across the patients. *SP100*, *RAD52* and *ATRX* get down-regulated in tumors.

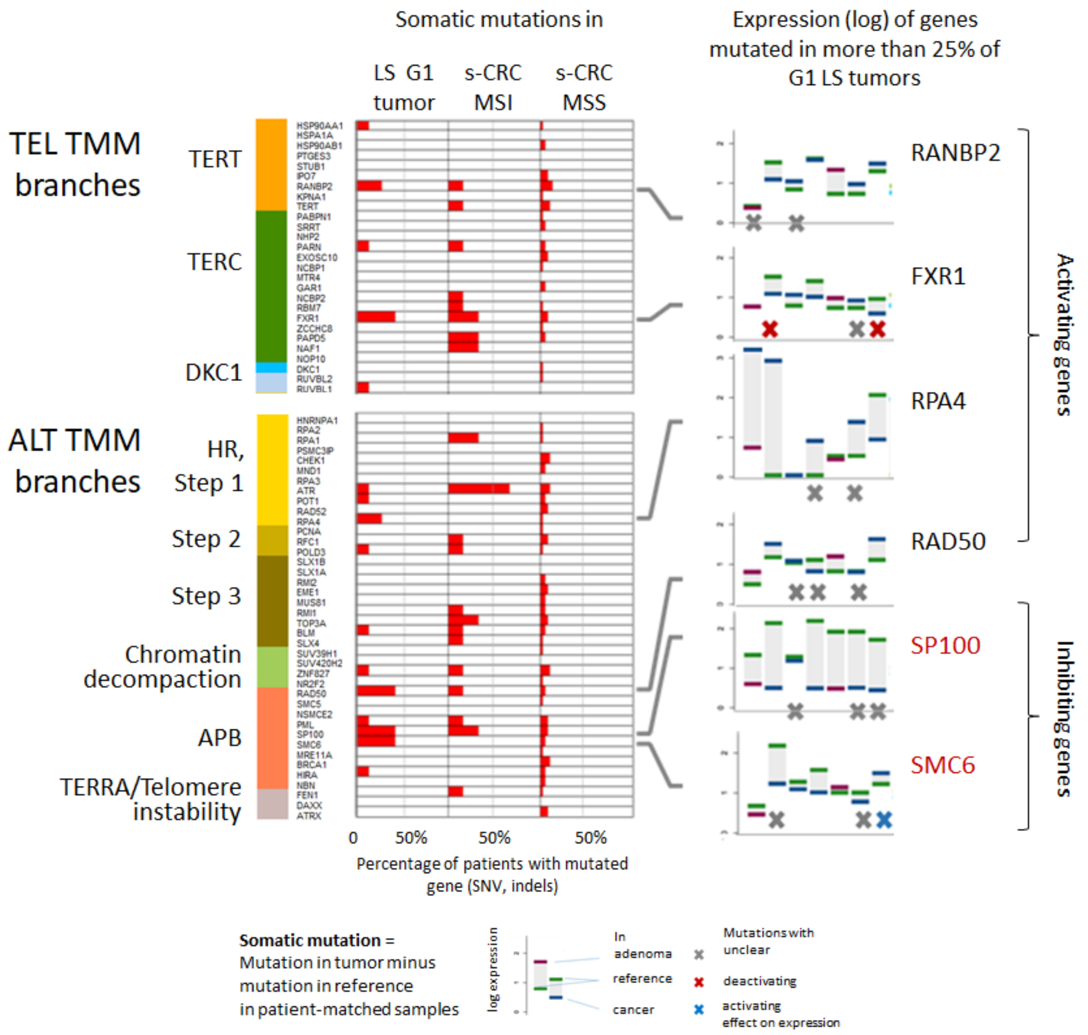

Supplementary Figure 10. Percentage of tumors with somatic mutations (SNV and indels) of TMM-genes in G1 LS-tumors and in MSI and MSS s-CRC (left part) and gene expression of recurrently mutated TMM genes in G1 LS-CRC samples (at minimum twice mutated in the seven tumors studied) (right part). The mutational load of the TMM genes in MSS s-CRC is markedly lower than in the hypermutated MSI s-CRC and G1 LS tumors, as expected from the overall mutational load (1). No recurrently mutated genes in more than 10% of the MSS s-CRC samples were detected, while in MSI s-CRC, ATR is mutated in more than 50% of the samples and further six genes are mutated in 40% (*FXR1*, *PAPD5*, *NAF1*, *RPA1*, *TOP3*, *SP100*). In G1 LS-tumors we found four genes (*FXR1*, *RAD50*, *SP100*, *SMC6*) mutated in 50% of the cancer samples (see also the crosses assigning the mutations to the patients in the right part of the figure). Overall the comparison of the expression of mutated and non-mutated genes and their changes with respect to reference mucosa shows no clear effect of the mutations except for *FXR1*, where two out of three cancer samples reverse the differential expression with respect to the reference what suggests a deactivating effect of the mutation. Overall we find recurrent mutations of the four genes in G1 LS-CRC and MSI s-CRC.

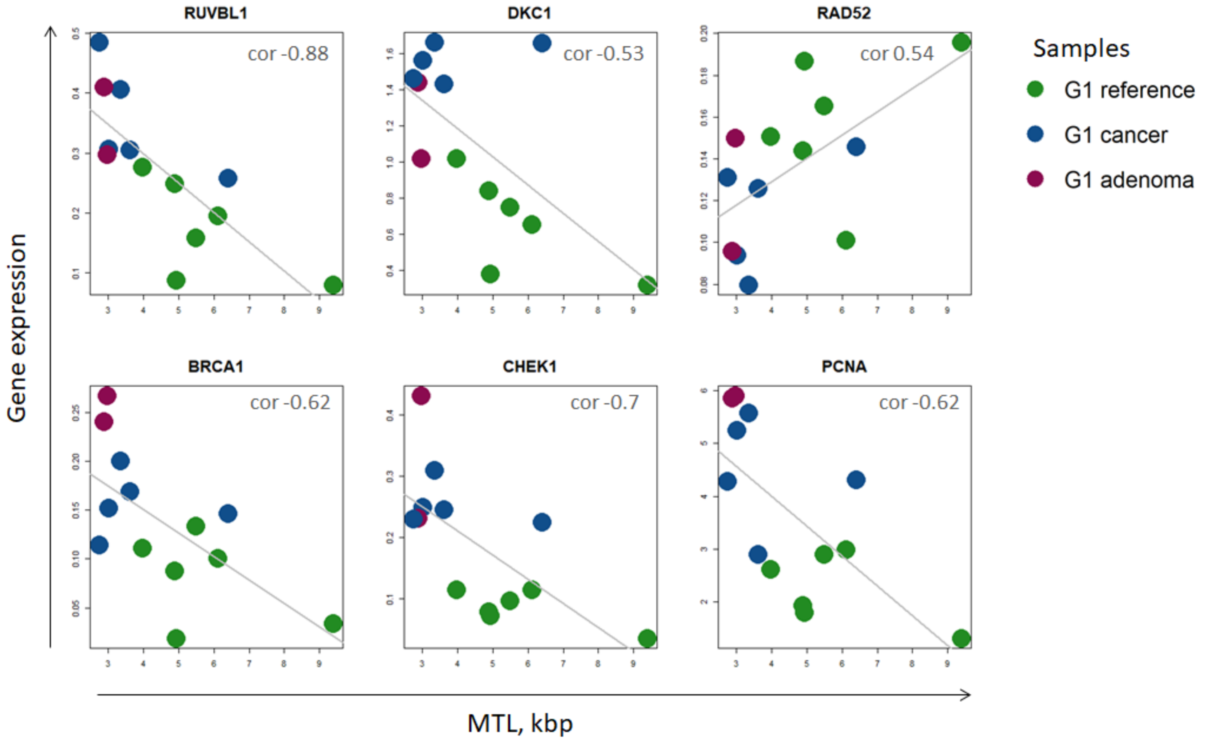

Supplementary Figure 11. Top genes showing largest Pearsons correlation coefficients (|cor|> 0.5, p< 0.05) between mean telomere length (MTL) and gene expression in G1 LS-CRC. Except for RAD52, all the genes are up-regulated with telomere shortening giving rise to negative correlations. The strongest correlation is observed for *RUVBL1* (pontin). Note also that gene expression changes in tumors account for about 50% of total variation while the respective MTL are virtually invariant. This difference reflects the asymptotic leveling off of MTL towards a critical limiting value in tumors where changes of gene expression of TMM genes associate with only minor attrition of MTL. A similar relationship was found for cell cycle activity (see main paper).

2.4 Partial influences of TMM genes

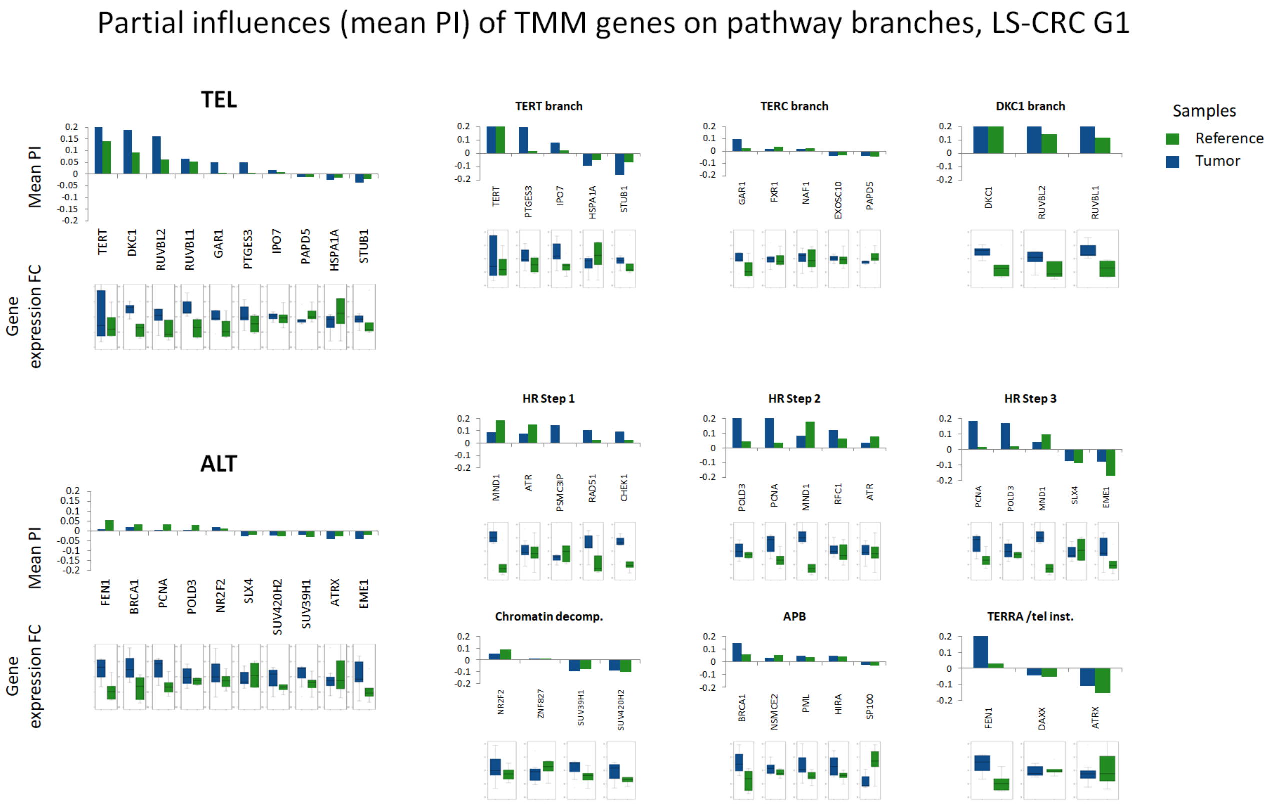

Supplementary Figure 12. Influences of TMM genes on TEL and ALT pathways in G1 LS-CRC: TEL-TMM is governed by *TERT*, *DKC1* and *RUVBL2*. The effect of *DKC1* is observed consistently in all tumors. ALT-TMM is affected by different genes in step 1-3 of the HR-branch.

Partial influences (mean PI) of TMM genes on pathway branches, s-CRC

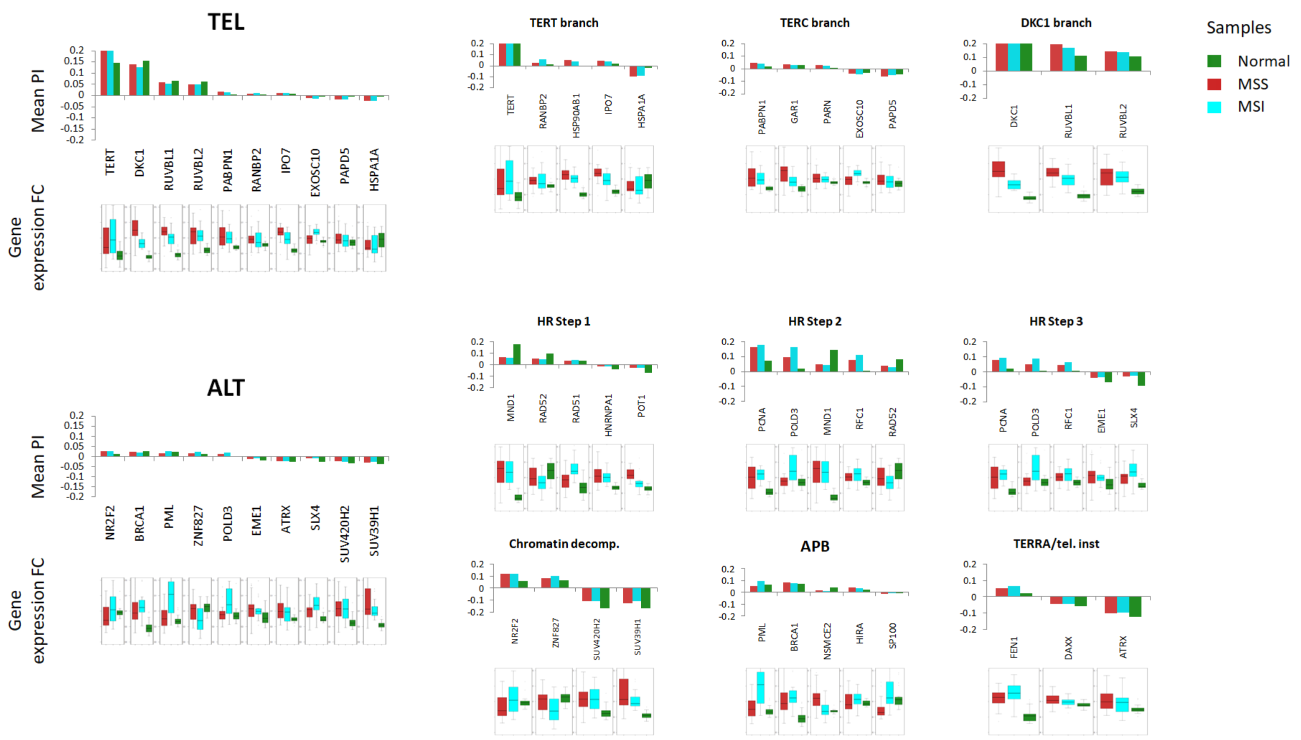

Supplementary Figure 13. Influences of TMM genes on TEL and ALT pathways in MSS/MSI s-CRC and in Normal mucosa.

3 Supplementary Tables

3.1 Supplementary Table 1. Genes included in TEL and ALT TMM pathways [a].

TMM-branch	Symbol	Name	Ref
TEL-TERT	HSP90AA1	heat shock protein 90 alpha family class A member 1	(13)
	HSPA1A	heat shock protein family A (Hsp70) member 1A	(14)
	HSP90AB1	heat shock protein 90 alpha family class B member 1	(13)
	PTGES3	prostaglandin E synthase 3	(13)
	STUB1	STIP1 homology and U-box containing protein 1	(14)
	IPO7	importin 7	(15)
	RANBP2	RAN binding protein 2	(15)
	KPNA1	karyopherin subunit alpha 1	(14)
	TERT	telomerase reverse transcriptase	(16)
TEL-TERC	PABPN1	poly(A) binding protein nuclear 1	(17)
	SRRT	serrate, RNA effector molecule	(17)
	NHP2	NHP2 ribonucleoprotein	(18)
	PARN	poly(A)-specific ribonuclease	(17)
	EXOSC10	exosome component 10	(17)
	NCBP1	nuclear cap binding protein subunit 1	(17)
	MTR4 (MTREX)	Mtr4 Exosome RNA Helicase	(17)
	GAR1	GAR1 ribonucleoprotein	(18)
	NCBP2	nuclear cap binding protein subunit 2	(17)
	RBM7	RNA binding motif protein 7	(17)
	FXR1	FMR1 autosomal homolog 1	(19)
	ZCCHC8	zinc finger CCHC-type containing 8	(17)
	PAPD5	poly(A) RNA polymerase D5, non-canonical	(17)
	NAF1	nuclear assembly factor 1 ribonucleoprotein	(18)
	NOP10	NOP10 ribonucleoprotein	(18)
TEL-DKC1	DKC1	dyskerin pseudouridine synthase 1	(18)
	RUVBL2	RuvB like AAA ATPase 2	(18)
	RUVBL1	RuvB like AAA ATPase 1	(18)
ALT-HR, Step1	HNRNPA1	heterogeneous nuclear ribonucleoprotein A1	(20)
	RPA2	replication protein A2	(20)
	RPA1	replication protein A1	(20)
	PSMC3IP	PSMC3 interacting protein	(21)
	CHEK1	checkpoint kinase 1	(22)
	MND1	meiotic nuclear divisions 1	(21)
	RPA3	replication protein A3	(20)
	ATR	ATR serine/threonine kinase	(22)
	POT1	protection of telomeres 1	(20)
	RAD52	RAD52 homolog, DNA repair protein	(20)
	RPA4	replication protein A4	(20)
ALT-HR, Step2	PCNA	proliferating cell nuclear antigen	(22)
	RFC1	replication factor C subunit 1	(22)
	POLD3	DNA polymerase delta 3, accessory subunit	(22)
ALT-HR, Step3	SLX1B	SLX1 homolog B, structure-specific endonuclease subunit	(20)

	SLX1A	SLX1 homolog A, structure-specific endonuclease subunit	(20)
	RMI2	RecQ mediated genome instability 2	(20)
	EME1	essential meiotic structure-specific endonuclease 1	(20)
	MUS81	MUS81 structure-specific endonuclease subunit	(20)
	RMI1	RecQ mediated genome instability 1	(20)
	TOP3A	topoisomerase (DNA) III alpha	(20)
	BLM	Bloom syndrome RecQ like helicase	(20)
	SLX4	SLX4 structure-specific endonuclease subunit	(20)
ALT-Chromatin decomapaction	SUV39H1	suppressor of variegation 3-9 homolog 1	(23)
	SUV420H2 (KMT5C)	Lysine Methyltransferase 5C	(23)
	ZNF827	zinc finger protein 827	(24)
	NR2F2	nuclear receptor subfamily 2 group F member 2	(24)
ALT-APB	RAD50	RAD50 double strand break repair protein	(25)
	SMC5	structural maintenance of chromosomes 5	(26)
	NSMCE2	NSE2/MMS21 homolog, SMC5-SMC6 complex SUMO ligase	(26)
	PML	promyelocytic leukemia	(27)
	SP100	SP100 nuclear antigen	(26)
	SMC6	structural maintenance of chromosomes 6	(26)
	MRE11A (MRE11)	MRE11 Homolog, Double Strand Break Repair Nuclease	(26)
	BRCA1	BRCA1, DNA repair associated	(28)
	HIRA	histone cell cycle regulator	(29)
	NBN	Nibrin	(26)
ALT-TERRA/Telomere instability	FEN1	flap structure-specific endonuclease 1	(30)
	DAXX	death domain associated protein	(31)
	ATRX	ATRX, chromatin remodeler	(31)

[a] For detailed description of the genes see (4,5) and http://big.sci.am/software/tmm/tmm-genes/

3.1 Supplementary Table 2: Verification of TMM genes using gene sets of the gene ontology (GO) category Biological Process (BP) and Cellular Component (CC)

For verification of the TMM genes we calculated enrichment of gene sets of independent functional assignments provided by the GO categories CC and BP taken from (7). Overall this analysis clearly indicates strong accumulation of genes with explicit relation to telomeric function on all levels of our TMM-pathway topologies (all TMM genes, TEL and ALT, all sub-branches, see table; gene sets with explicit relation to telomere biology are underlined). Moreover, enrichment analysis indicates extra-telomeric functions related, e.g. to PML bodies and different DNA-repair mechanisms, as considered in our TMM-pathways.

<table>
<tr><th>All TMM genes [a]</th><th>TEL and ALT TMM</th><th>TMM-Branches [a]</th></tr>
<tr><td rowspan="4">TEL&ALT:
CC:
PML body (-21)
Nucleoplasm (-17)
nuclear chromosome, telomeric (-15)
chromosome, telomeric region (-15)
nucleus(-10)
site of double-strand break (-8)
BP:
double-strand break repair via homologous recombination (-29); DNA repair (-26); telomere maintenance (-23); double-strand break repair (-22); DNA recombination (-17); telomere</td><td rowspan="3">TEL:
CC:
telomerase holoenzyme complex (-13); nucleoplasm (-07); small nucleolar ribonucleoprotein complex (-06); nuclear inclusion body (-06)
BP:
telomere maintenance via telomerase (-11); pseudouridine synthesis (-08); regulation of cellular response to heat (-07); rRNA processing (-06)</td><td>TEL-TERT:
CC: small nucleolar ribonucleoprotein complex (-08); telomerase holoenzyme complex (-07); mRNA cap binding complex (-05); nuclear chromosome, telomeric region (-3)
BP: regulation of cellular response to heat (-9); cellular response to heat (-08); regulation of protein ubiquitination (-07); protein import into nucleous (-06); telomere maintenance (-5)</td></tr>
<tr><td>TEL-TERC:
CC: nuclear pore (-05); nuclear inclusion body (-5); telomerase holoenzyme complex (-5)
BP: telomere maintenance via telomerase (-07); pseudouridine synthesis (-07)
rRNA processing (-06); nuclear-transcribed mRNA catabolic process, nonsense-mediated decay (-06); ribosome biogenesis (-06)
mRNA 3'-end processing (-06)</td></tr>
<tr><td>TEL-DKC1:
CC: Ino80 complex (-6), NuA4 histone acetyltransferase complex (-6), MLL1 complex (-6); nuclear matrix (-5); telomerase holoenzyme complex (-3)
BP: histone H2A acetylation (-06); histone H4 acetylation (-06); DNA duplex unwinding (-05); regulation of growth (-05); DNA recombination (-05)</td></tr>
<tr><td>ALT:
CC:
PML body (-21); Nucleoplasm (-17); nuclear chromosome, telomeric region(-15); chromosome,</td><td>ALT-HR1:
CC: DNA replication factor A complex (-11); nucleoplasm (-6); nuclear chromosome, telomeric region (-5); chromosome, telomeric region (-5); PML body (-5)
BP:DNA recombination (-14); double-strand break repair via homologous recombination (-10); double-strand break repair (-10); DNA replication (-10); DNA repair (-09); interstrand cross-link repair (-08)</td></tr>
</table>

maintenance via recombination (-17)	telomeric region(-15); nucleus(-10); site of double-strand break(-08)<br>**BP:**<br>double-strand break repair via homologous recombination (-35); double-strand break repair (-28); DNA repair (-28); telomere maintenance via recombination (-19); DNA replication (-18); telomere maintenance (-18)	**ALT-HR2:**<br>CC: Nucleoplasm (-3); nuclear chromosome, telomeric region (-2)<br>BP: nucleotide-excision repair, DNA gap filling (-09); telomere maintenance via semi-conservative replication (-09); telomere maintenance via recombination (-09): DNA strand elongation involved in DNA replication (-09); DNA damage response, detection of DNA damage (-08); nucleotide-excision repair, DNA incision (-08)
		**ALT-HR3:**<br>CC: Nucleoplasm (-7); PML body (-3); nuclear chromatin (-3)<br>BP: double-strand break repair via homologous recombination (-18); double-strand break repair (-18); DNA repair (-14); interstrand cross-link repair (-11); double-strand break repair via synthesis-dependent strand annealing (-09)<br>nucleic acid phosphodiester bond hydrolysis (-07)
		**ALT-ALT-ChrD:**<br>CC: Heterochromatin (-3); condensed nuclear chromosome (-2)<br>BP: transcription, DNA-templated(-3)
		**ALT-ApB:**<br>CC: PML body (-15); chromosome, telomeric region (-10); site of double-strand break (-10); nucleoplasm (-8); nuclear chromosome, telomeric region (-6)<br>BP: protein sumoylation (-12); double-strand break repair via homologous recombination (-11); cellular senescence (-10); post-translational protein modification (-08); cellular response to DNA damage stimulus (-08); double-strand break repair via synthesis-dependent strand annealing (-07)
		**ALT-TTI**:<br>CC: nuclear chromosome, telomeric region (-4); PML body (-3); pericentric heterochromatin (-2)<br>BP: chromatin remodeling (-4); nucleosome assembly (-3)

[a] Gene sets are taken from gene ontology categories cellular component (CC) and biological process (BP). Enrichment p-values for accumulation of gene sets in the TMM-genes were calculated using Fisher's exact test. The enrichment p-value is provided as decadic logarithm in brackets (e.g. -7 means $p= 10^{-7}$). Gene sets with explicit relation to telomeric functions are underlined.

3.2 Supplementary Table 3: Verification of TMM genes using TelNet database information (8) [a]

TelNet collects about 2,000 genes with impact for telomere biology and provided different categories of relevance, namely cell and telomere maintenance (TM) function, TM-annotation (either to TEL, ALT or ambiguous mechanisms), significance extracted from literature knowledge (validated, predicted, screened) and an ‘importance’ score ranging from 1 (low) to 10 (very high). Part A of the table lists the relative entries of TelNet for the TMM genes used in this publication. Part B provides selected overview statistics which compare the TelNet entries with our TMM annotations. Overall this analysis confirms the TMM-gene functions and their relevance for telomere biology with respect to TEL- and ALT-mechanisms. It also indicates extra-telomeric functions of the genes. Part A of the table shows that typically the majority of genes in each of our pathways were confirmed by TelNet in terms of TM-mechanism (TEL or ALT), experimental validation and importance scoring. Part B of the table revealed that genes with crucial importance for telomere maintenance clearly accumulate in our TMM-pathways compared with the gene reservoir collected in TelNet. Note that we understand TelNet as an independent data source to evaluate gene’s relevance for telomere biology but not as ‘gold standard’. We interpret TelNet categories such as ‘TM annotation’, ‘TM-significance’ and ‘TelNet-score’ as curated information about possible roles of genes in telomere biology extracted from literature, which however requires detailed review and further evaluation for each particular gene.

A) Gene characteristics

Gene [a]	Cell functions [b]	TM functions [b]	TM annotation [b]	effect	TM-significance [b]	TelNet score [b]
**TEL-TERT** [c]				**67% TEL**	**33%** [c]	**4.0** [c]
**HSP90AA1**	Chromatin organization	Gene regulation; Telomerase activity;	ALT: ambiguous; TEL: enhancing	TEL	validated	**6**
**HSPA1A**	Cell membrane / wall		ALT: ambiguous; TEL: ambiguous	ambiguous	screened	**2**
**HSP90AB1**	Chromatin organization	Telomerase activity;	ALT: ambiguous; TEL: enhancing	TEL	screened	**2**
**PTGES3**	Protein synthesis and degradation	Telomerase activity;	ALT: ambiguous; TEL: enhancing	TEL	predicted	**2**

**STUB1**	DNA replication and repair		ALT: ambiguous; TEL: ambiguous	ambigu ous	screened	**2**
**TERT** [d]	Telomere biology	Telomerase component; Telomere length; Telomerase activity; Telomere repeat binding;	ALT: repressing; TEL: enhancing	TEL	validated	**10**
**TEL-TERC** [d]				**67% TEL**	**17%**	**2.8**
**NHP2**	RNA transcription and processing	Telomerase activity;	ALT: ambiguous; TEL: enhancing	TEL	screened	**3**
**NCBP1**	--- not yet entered ---		ALT: ambiguous; TEL: ambiguous	ambigu ous	predicted	**1**
**GAR1**	RNA transcription and processing	Telomerase activity;	ALT: ambiguous; TEL: enhancing	TEL	validated	**6**
**NCBP2**	RNA transcription and processing		ALT: ambiguous; TEL: ambiguous	ambigu ous	predicted	**1**
**NAF1**	RNA transcription and processing	Telomere length; Telomerase activity;	ALT: ambiguous; TEL: enhancing	TEL	screened	**3**
**NOP10**	RNA transcription and processing	Telomerase activity;	ALT: ambiguous; TEL: enhancing	TEL	screened	**3**
**TEL-DKC1**				**100% TEL**	**100%**	**6.0**
**DKC1**	Telomere biology	Telomerase activity;	ALT: ambiguous; TEL: enhancing	TEL	validated	**7**
**RUVBL2**	DNA replication and repair	Telomerase activity; Gene regulation;	ALT: ambiguous; TEL: enhancing	TEL	validated	**6**
**RUVBL1**	Chromatin organization	Telomerase activity;	ALT: ambiguous; TEL: enhancing	TEL	validated	**5**
**ALT-HR1**				**50% ALT**	**50%**	**5.0**

**HNRNPA1**	RNA transcription and processing	Telomere repeat binding; Telomerase activity;	ALT: ambiguous; TEL: repressing	ALT	predicted	**2**
**RPA2**	DNA replication and repair	DNA repair / damage response; ALT associated PML nuclear bodies (APBs); DNA recombination; TERRA;	ALT: ambiguous; TEL: ambiguous	ambiguous	validated	**7**
**RPA1**	DNA replication and repair	DNA repair / damage response; ALT associated PML nuclear bodies (APBs); DNA recombination;	ALT: repressing; TEL: ambiguous	ALT repr.	validated	**7**
**CHEK1**	Cell cycle	DNA repair / damage response; Telomerase activity;	ALT: ambiguous; TEL: enhancing	TEL	screened	**3**
**RPA3**	DNA replication and repair	DNA repair / damage response; DNA recombination;	ALT: enhancing; TEL: ambiguous	ALT	predicted	**2**
**ATR**	DNA replication and repair	DNA repair / damage response; Telomerase activity; ALT associated PML nuclear bodies (APBs); Telomerase localization;	ALT: enhancing; TEL: enhancing	ambiguous	validated	**7**
**POT1**	Telomere biology	Shelterin component; Telomere repeat binding; Telomere structure; Telomerase activity; ALT associated PML nuclear bodies (APBs)	ALT: repressing; TEL: enhancing	ALT repr.	validated	**10**
**RAD52**	DNA replication and repair	DNA repair / damage response; ALT associated PML nuclear bodies (APBs);	ALT: ambiguous; TEL: ambiguous	ambiguous	predicted	**2**
**ALT-HR2**				**67% ALT**	**67%**	**5.3**
**PCNA**	Cell cycle	DNA repair / damage response; ALT associated PML nuclear bodies (APBs); DNA replication; DNA recombination;	ALT: ambiguous; TEL: ambiguous	ambiguous	validated	**7**
**RFC1**	DNA replication and repair	DNA replication; Telomerase activity; DNA recombination;	ALT: enhancing; TEL: ambiguous	ALT	predicted	**3**

**POLD3**	DNA replication and repair	DNA replication; DNA recombination; Extrachromosomal telomeric repeat formation; Telomere length;	ALT: enhancing; TEL: ambiguous	ALT	validated	**6**
**ALT-HR3**				**63% ALT**	**38%**	**3.8**
**SLX1A**	DNA replication and repair	Telomere structure; Extrachromosomal telomeric repeat formation;	ALT: enhancing; TEL: ambiguous	ALT	predicted	**2**
**RMI2**	DNA replication and repair	DNA replication;	ALT: ambiguous; TEL: ambiguous	ambigu ous	screened	**2**
**EME1**	DNA replication and repair	DNA repair / damage response;	ALT: ambiguous; TEL: ambiguous	ambigu ous	predicted	**2**
**MUS81**	DNA replication and repair	DNA repair / damage response; ALT associated PML nuclear bodies (APBs); DNA recombination;	ALT: enhancing; TEL: ambiguous	ALT	validated	**6**
**RMI1**	DNA replication and repair	DNA replication;	ALT: ambiguous; TEL: ambiguous	ambigu ous	screened	**2**
**TOP3A**	DNA replication and repair	DNA recombination; ALT associated PML nuclear bodies (APBs);	ALT: enhancing; TEL: ambiguous	ALT	validated	**6**
**BLM**	DNA replication and repair	ALT associated PML nuclear bodies (APBs); DNA repair / damage response; Telomere structure; Extrachromosomal telomeric repeat formation; Shelterin binding	ALT: enhancing; TEL: ambiguous	ALT	validated	**8**
**SLX4**	DNA replication and repair	Extrachromosomal telomeric repeat formation;	ALT: enhancing; TEL: ambiguous	ALT	screened	**2**
**ALT-ChrD**				**75% ALT**	**50%**	**4.25**
**SUV39H1**	Chromatin organization	Histone modification / variants; Chromatin structure;	ALT: ambiguous; TEL: ambiguous	ambigu ous	predicted	**2**
**SUV420H2**	Chromatin organization	Histone modification / variants; ALT associated PML nuclear bodies (APBs);	ALT: enhancing; TEL: ambiguous	ALT	screened	**3**

**ZNF827**	--- unknown ---		ALT: enhancing; TEL: ambiguous	ALT	validated	**5**
**NR2F2**	RNA transcription and processing	ALT associated PML nuclear bodies (APBs); Telomerase activity; Gene regulation;	ALT: enhancing; TEL: repressing	ALT	validated	**7**
**ALT-APB**				**88% ALT**	**89%**	**6.8**
**RAD50**	DNA replication and repair	ALT associated PML nuclear bodies (APBs); DNA repair / damage response; DNA recombination; Telomere repeat binding; DNA replication	ALT: enhancing; TEL: repressing	ALT	validated	**9**
**SMC5**	DNA replication and repair	DNA repair / damage response; DNA recombination;	ALT: enhancing; TEL: ambiguous	ALT	validated	**5**
**NSMCE2**	Protein modification	Sumoylation; ALT associated PML nuclear bodies (APBs); DNA repair / damage response; DNA recombination;	ALT: enhancing; TEL: ambiguous	ALT	validated	**7**
**PML**	Nuclear organization	ALT associated PML nuclear bodies (APBs); Sumoylation; DNA repair / damage response;	ALT: enhancing; TEL: ambiguous	ALT	validated	**9**
**SP100**	Nuclear organization	ALT associated PML nuclear bodies (APBs);	ALT: enhancing; TEL: ambiguous	ALT	validated	**6**
**SMC6**	DNA replication and repair	DNA repair / damage response; DNA recombination;	ALT: enhancing; TEL: ambiguous	ALT	validated	**6**
**MRE11A**	DNA replication and repair	DNA recombination; Telomerase activity; ALT associated PML nuclear bodies (APBs); DNA repair / damage response; DNA replication	ALT: enhancing; TEL: ambiguous	ALT	validated	**8**
**BRCA1**	DNA replication and repair	DNA repair / damage response;	ALT: ambiguous; TEL: ambiguous	ambigu ous	predicted	**2**

**NBN**	Cell cycle	DNA repair / damage response; ALT associated PML nuclear bodies (APBs); DNA replication; Extrachromosomal telomeric repeat formation; Shelterin binding	ALT: enhancing; TEL: ambiguous	ALT	validated	**9**
**ALT-TTCI**				**100% ALT**	**100%**	**6.7**
**FE1**	A replication and repair	DNA repair / damage response; ALT associated PML nuclear bodies (APBs); DNA replication; DNA recombination;	ALT: enhancing; TEL: ambiguous	ALT	validated	**8**
**DAXX**	Chromatin organization	Protein folding; Histone assembly;	ALT: repressing; TEL: ambiguous	ALT repr.	validated	**6**
**ATRX**	Chromatin organization	Chromatin remodeling; Telomere structure; Telomere repeat binding;	ALT: repressing; TEL: ambiguous	ALT repr.	validated	**6**

[a] TelNet included the following: (i) Proteins that were purified with a telomere probe in an ALT- and a telomerase-positive cell line, (ii) proteins from the analysis of telomeric chromatin of telomerase-positive cells, (iii) proteins in close proximity to shelterin components, (iv) proteins that affected ALT-associated PML nuclear bodies, (v) deregulated proteins linked to telomere shortening, (vi) genes identified from telomerase activity signatures derived from gene expression data, (vii) telomerase regulators identified in a kinase screen and transcription factors compiled in a review and, (viii) a gene set with potential relevance to telomeres and the ALT pathway.

[b] **TelNet terms:**

Cellular Function and TM function: Every gene was manually annotated with the respective term that was most representative for its cellular function. In this manner, general information for every gene entry was compiled from a variety of external databases.

TMM annotation: Information on the protein's activity. It distinguished between “alternative lengthening of telomeres (ALT)” versus “telomerase-mediated” regulation with the associated activities “repressing”, “enhancing” or “ambiguous”. The latter refers to cases where literature information was inconsistent or was used for genes that were mentioned in the context of ALT or telomerase without further details of regulation activity.

TM significance: screened (if gene is mentioned in screening studies), predicted (if gene has suggested role in telomere biology without experimental validation), validated (…with experimental validation)

TM score: To quantify the significance of a given gene for TM TelNet scores each gene ranging from 1 (low) to 10 (high) that was automatically calculated from information entered into the TelNet database. Scoring criteria included the cellular function, number and relevance of assigned TM functions and the amount of experimental data associated with the TM function of a given gene.

[c] TEL and ALT branches are marked with light-apricot and -blue background, respectively. Percentage of genes assigned to TEL or ALT were counted for each of the TEL- and ALT-TMM subbranches of our TMM-pathways. TM significance and TelNet-score are provided as % of validated genes and mean value of the genes of the respective branch.

[d] Genes with cellular function ‘telomere biology’ are indicated by light-grey background

B) Comparison of TelNet statistics with that of TMM genes used in this work

TelNet statistics (see also https://malone2.bioquant.uni-heidelberg.de/fmi/webd/TelNet):

	**TelNet**	**TMM pathways in this work**
Total number of genes in	2093	67
**Genes with TelNet-score > 6 [a]**	**45 (2%)**	**14 (21%)**
**Genes with TelNet score > 4**	**164 (8%)**	**27 (40%)**
**Validated genes**	**165 (8%)**	**28 (40%)**
**Genes with cellular function ‘Telomere Biology’**	**26 (1%)**	**3 (4%)**
**Genes with cellular function ‘DNA replication and repair’**	**222 (11%)**	**21 (31%)**
Genes with cellular function ‘RNA transcription and processing’	255 (12%)	7 (10%)
Genes with cellular function ‘Nuclear Organization’	9 (4 %)	2 (3%)
Genes with cellular function ‘Chromatin organization’	172 (8%)	6 (9%)

Genes with cellular function ’Cell Cycle	80 (4%)	4 (6%)
**Genes with TM function ‘Telomerase activity’**	**298 (14%)**	**15 (22%)**
**Genes with TM function ‘DNA repair, damage response’**	**134 (6%)**	**18 (27%)**
**Genes with TM function ‘ALT associated PML’**	**71 (3%)**	**17 (27%)**
**Genes with TM function ‘Telomere length’**	**30 (1%)**	**3 (4%)**
**TMM annotation TEL enhancing/ ALT ambiguous**	**172 (8%)**	**13 (19%)**
**TMM annotation ALT enhancing/ TEL ambiguous**	**76 (3%)**	**23 (34%)**
TMM annotation ALT ambiguous/ TEL repressing	69 (3%)	3 (4%)

[a] Gene categories which are enriched in the TMM-pathways used in this work compared with TelNet were highlighted with bold letters.

3.3 Supplementary Table 4: Telomere and TRV length analysis

A) Telomere shortening in tumors compared with reference

Mean MTL-differences (ΔMTL, tumor minus reference) and p-values indicate significant ($p<0.05$) shortening of telomeres for LS-CRC and s-CRC. For different subtype-strata p-values mostly exceed 0.05.

Subtype	ΔMTL / bp	W p value [a]	t test p [b]
**All LS-CRC**	-2211	0,024	0,011
**LS-CRC G1**	-2108	0,219	0,134
**LS-CRC G2**	-2335	0,125	0,045
**All s-CRC**	-2612	0,002	0,008
**s-CRC MSI**	-3329	0,016	0,029
**s-CRC MSS**	-1466	0,188	0,140

[a] paired Wilcoxon signed-rank test
[b] paired t-test

A) Mean cumulative TRV length differences (ΔMCL) between MSI and MSS s-CRC

TRV are systematically longer in MSI s-CRC compared with MSS s-CRC. The table lists 6 TRV meeting the significance threshold ($p< 0.05$) and further 6 ($p< 0.1$) and 7 ($p< 0.2$) TRV taken from the p-ranked list. Four TRV associate with differential features ALT-versus TEL TMM according to previous reports (see comments). Three of them in addition show largest ΔMCL values among all TRVs studied by us. Overall these results suggest gradual differences in TRV abundance between TEL and ALT TMM between MSI and MSS s-CRC with a trend towards ALT-associated relative TRV composition in MSI.

TRV[c]	ΔMCL / bp	W p[a]	t-test[b] p	comment
TTAGGG	422	0,5	0,6	canonical repeat
TTATGGG	0,10	0,01	0,02	
**TTCGGG**	**2,20**	**0,02**	**0,02**	One of the top TRVs in ALT classifier (32), induced by TEL (12)
AGGGGT	0,24	0,02	0,04	
TAGGGG	0,84	0,05	0,08	
**TCAGGG**	**1,83**	**0,05**	**0,04**	Cell line-specific enrichment in ALT (12), among the TRVs in ALT classifier (32)
GCTGGG	0,03	0,05	0,03	

TTAGGT	0,34	0,07	0,26	
AGGGTT	0,19	0,07	0,06	
**TTGGGG**	**1,94**	**0,09**	**0,08**	ALT-related, abolishes shelterin binding (32–34)
TTAGG	0,36	0,09	0,12	
TTTTAGGG	0,37	0,09	0,06	
AAAGTG	-0,01	0,09	0,14	
TTAAGG	0,39	0,13	0,11	Classifies ALT-vs-TEL (32)
TTTAGGG	1,07	0,17	0,12	
TTGGGT	0,06	0,17	0,14	
GGGTTG	0,03	0,17	0,23	
ACTCAT	-0,02	0,17	0,17	
TTACGG	0,10	0,17	0,17	
AAGGGT	0,09	0,17	0,19	

[c] TRV are ranked with decreasing significance. Only cumulative TRV length differences (ΔMCL) providing p<0.2 were considered. Horizontal lines refer to p<0.05 and p<0.1, respectively. Canonical repeats (no significant length difference between MSI and MSS) are shown as first row. Largest TRV-lengths were highlighted with bold letters.

Supplementary Table 5. Differential expression of TMM genes in CRC

G1 LS-CRC				MSS s-CRC				MSI s-CRC			
**Gene**	**log2 FC**	**p value**	**padj**	**Gene**	**log2 FC**	**p value**	**padj**	**Gene**	**log2 FC**	**p value**	**padj**
SP100	-1.58	0	0	PTGES3	0.50	0.00	0.00	CHEK1	1.39	0.00	0.00
NHP2	1.92	0	0	RUVBL2	0.99	0.00	0.00	STUB1	0.16	0.19	0.28
CHEK1	1.75	0	0	STUB1	0.07	0.49	0.56	HSP90AB1	1.08	0.00	0.00
RUVBL2	1.74	0	0	NSMCE2	0.93	0.00	0.00	MND1	1.86	0.00	0.00
DKC1	1.21	0	0	DKC1	1.86	0.00	0.00	PTGES3	0.53	0.00	0.00
GAR1	1.56	0	0	CHEK1	1.31	0.00	0.00	RUVBL2	0.88	0.00	0.00
EME1	2.06	0	0	ATR	0.82	0.00	0.00	ZNF827	-0.46	0.02	0.03
SUV420H2	1.31	0	0.001	MND1	1.73	0.00	0.00	BRCA1	1.34	0.00	0.00
MND1	2.48	0	0.001	NOP10	0.09	0.45	0.53	SP100	0.18	0.27	0.36
HSP90AB1	0.86	0	0.001	PARN	0.31	0.00	0.00	NOP10	0.36	0.01	0.02
HSP90AA1	1.07	0	0.001	BRCA1	1.32	0.00	0.00	NCBP1	-0.13	0.26	0.35
FEN1	1.62	0	0.002	RAD51	0.54	0.00	0.00	PCNA	1.11	0.00	0.00
PTGES3	0.95	0	0.003	POLD3	0.10	0.43	0.51	DKC1	1.16	0.00	0.00
PCNA	1.17	0	0.003	NAF1	0.48	0.00	0.00	EME1	0.41	0.02	0.04
RUVBL1	1.07	0	0.003	NR2F2	-0.02	0.90	0.93	RPA3	0.60	0.00	0.00
BRCA1	1.25	0.001	0.007	NCBP1	0.06	0.53	0.60	RPA1	0.30	0.00	0.00
HNRNPA1	0.77	0.001	0.007	RPA1	-0.22	0.00	0.01	POLD3	0.55	0.00	0.00
HIRA	0.75	0.001	0.008	ZNF827	-0.06	0.71	0.77	PAPD5	0.13	0.33	0.43
RAD51	1.91	0.002	0.012	HSP90AB1	1.25	0.00	0.00	RAD50	0.14	0.34	0.44
IPO7	0.96	0.005	0.023	EME1	0.43	0.00	0.01	SUV39H1	0.92	0.00	0.00
SUV39H1	0.92	0.009	0.033	PCNA	1.05	0.00	0.00	NAF1	0.43	0.00	0.00
RMI2	1.49	0.016	0.054	SMC6	0.35	0.00	0.00	BLM	1.11	0.00	0.00
MTR4	-0.64	0.031	0.087	RAD50	0.45	0.00	0.00	**TERT**	1.45	0.00	0.00
RPA3	0.9	0.031	0.088	FXR1	0.56	0.00	0.00	POT1	0.54	0.00	0.00
POLD3	0.54	0.043	0.112	RPA4	1.21	0.00	0.00	RMI1	0.47	0.00	0.00
STUB1	0.69	0.057	0.139	SUV39H1	1.14	0.00	0.00	KPNA1	0.10	0.22	0.30
NOP10	0.82	0.082	0.18	NBN	0.75	0.00	0.00	FXR1	0.42	0.00	0.01
NSMCE2	0.31	0.097	0.203	GAR1	0.84	0.00	0.00	MTR4	-0.27	0.02	0.04
NCBP2	0.27	0.103	0.213	**TERT**	1.43	0.00	0.00	GAR1	0.50	0.00	0.00
SRRT	0.4	0.108	0.222	IPO7	1.05	0.00	0.00	RFC1	0.40	0.00	0.00
**TERT**	**1.45**	**0.116**	**0.233**	KPNA1	0.14	0.04	0.07	NR2F2	0.26	0.23	0.32
ATRX	-0.74	0.127	0.248	RMI1	0.26	0.03	0.04	RPA4	0.84	0.02	0.04
ZNF827	-0.46	0.149	0.28	PABPN1	0.70	0.00	0.00	FEN1	1.23	0.00	0.00
EXOSC10	0.23	0.164	0.299	ATRX	0.64	0.00	0.00	RAD51	0.99	0.00	0.00
RAD52	-0.38	0.185	0.326	HNRNPA1	0.79	0.00	0.00	ATR	0.36	0.00	0.00
RBM7	0.37	0.209	0.355	MRE11A	1.17	0.00	0.00	NSMCE2	0.21	0.21	0.30
TOP3A	0.23	0.231	0.381	HSPA1A	0.21	0.40	0.48	TOP3A	0.54	0.00	0.00
SMC6	-0.33	0.242	0.393	RFC1	0.32	0.00	0.00	RAD52	-0.52	0.00	0.00

RMI1	0.41	0.255	0.408	SMC5	-0.22	0.03	0.05	RPA2	0.70	0.00	0.00
PABPN1	0.22	0.264	0.418	RPA3	0.79	0.00	0.00	IPO7	0.69	0.00	0.00
PML	0.41	0.28	0.437	EXOSC10	0.20	0.03	0.05	NHP2	0.68	0.00	0.00
POT1	-0.36	0.304	0.462	PAPD5	0.39	0.00	0.00	HSPA1A	0.27	0.35	0.45
RAD50	0.28	0.341	0.5	POT1	0.98	0.00	0.00	PARN	0.19	0.02	0.04
DAXX	-0.17	0.362	0.522	MUS81	-0.02	0.85	0.88	DAXX	0.17	0.07	0.11
NAF1	0.28	0.377	0.537	SUV420H2	0.79	0.00	0.00	ZCCHC8	0.14	0.07	0.12
SMC5	-0.23	0.48	0.63	RPA2	0.43	0.00	0.00	MUS81	-0.02	0.87	0.91
ATR	-0.25	0.494	0.642	PML	0.39	0.01	0.02	HNRNPA1	0.68	0.00	0.00
SLX4	-0.2	0.577	0.712	SP100	-0.39	0.00	0.01	NBN	0.71	0.00	0.00
HSPA1A	0.3	0.578	0.713	SRRT	0.52	0.00	0.00	PABPN1	0.56	0.00	0.00
BLM	0.33	0.637	0.76	TOP3A	-0.11	0.29	0.37	ATRX	0.34	0.02	0.04
FXR1	-0.12	0.642	0.763	RAD52	-0.28	0.03	0.04	RBM7	0.06	0.67	0.75
MUS81	-0.11	0.655	0.773	RMI2	0.76	0.00	0.00	HIRA	0.23	0.10	0.16
RPA4	-0.51	0.659	0.776	MTR4	-0.13	0.19	0.25	RANBP2	0.32	0.01	0.02
KPNA1	-0.09	0.714	0.817	HSP90AA1	0.91	0.00	0.00	SMC6	0.00	0.97	0.98
PAPD5	-0.08	0.729	0.827	ZCCHC8	0.02	0.76	0.80	HSP90AA1	1.31	0.00	0.00
RANBP2	0.13	0.729	0.828	SLX1A	-0.18	0.29	0.36	SLX1A	-0.25	0.22	0.30
PARN	-0.06	0.763	0.85	SLX4	0.42	0.00	0.00	SLX4	0.77	0.00	0.00
ZCCHC8	0.07	0.774	0.857	HIRA	0.12	0.29	0.37	HP1	0.11	0.51	0.61
SLX1A	0.18	0.781	0.862	BLM	1.15	0.00	0.00	NCBP2	0.29	0.01	0.03
NR2F2	0.16	0.786	0.865	PSMC3IP	-0.11	0.34	0.42	SUV420H2	0.88	0.00	0.00
NBN	0.09	0.806	0.879	RUVBL1	1.44	0.00	0.00	EXOSC10	0.43	0.00	0.00
SLX1B	0.16	0.812	0.883	FEN1	0.98	0.00	0.00	MRE11A	0.46	0.00	0.00
RPA2	-0.06	0.818	0.886	NHP2	0.95	0.00	0.00	RUVBL1	1.13	0.00	0.00
MRE11A	0.09	0.838	0.899	RANBP2	0.38	0.00	0.00	RMI2	0.85	0.00	0.00
RPA1	-0.05	0.847	0.904	NCBP2	0.82	0.00	0.00	PML	1.19	0.00	0.00
RFC1	-0.06	0.85	0.906	DAXX	0.31	0.00	0.00	SRRT	0.54	0.00	0.00
NCBP1	-0.02	0.892	0.935	RBM7	-0.26	0.02	0.04	SLX1B	-0.25	0.22	0.30
PSMC3IP	0.05	0.915	0.949	SLX1B	-0.19	0.27	0.35	PSMC3IP	0.01	0.96	0.98
HP1	0	1	1	HP1	0.39	0.01	0.01	SMC5	-0.08	0.50	0.60

4 References

1. Binder H, Hopp L, Schweiger MR, Hoffmann S, Jühling F, Kerick M, Timmermann B, Siebert S, Grimm C, Nersisyan L, et al. Genomic and transcriptomic heterogeneity of colorectal tumours arising in Lynch syndrome. *J Pathol* (2017) **243**:242–254. doi:10.1002/path.4948

2. Luo Z, Wang W, Li F, Songyang Z, Feng X, Xin C, Dai Z, Xiong Y. Pan-cancer analysis identifies telomerase-associated signatures and cancer subtypes. *Mol Cancer* (2019) **18**:106. doi:10.1186/s12943-019-1035-x

3. Barthel FP, Wei W, Tang M, Martinez-Ledesma E, Hu X, Amin SB, Akdemir KC, Seth S, Song X, Wang Q, et al. Systematic analysis of telomere length and somatic alterations in 31 cancer types. *Nat Genet* (2017) **49**:349–357. doi:10.1038/ng.3781

4. Nersisyan L, Arakelyan A. A transcriptome and literature guided algorithm for reconstruction of pathways to assess activity of telomere maintenance mechanisms. *bioRxiv* (2017)200535. doi:10.1101/200535

5. Nersisyan L. Telomere Analysis Based on High-Throughput Multi -Omics Data. (2017) Available at: urn:nbn:de:bsz:15-qucosa2-162974

6. Lafferty-Whyte K, Cairney CJ, Will MB, Serakinci N, Daidone M-G, Zaffaroni N, Bilsland A, Keith WN. A gene expression signature classifying telomerase and ALT immortalization reveals an hTERT regulatory network and suggests a mesenchymal stem cell origin for ALT. *Oncogene* (2009) **28**:3765–3774. doi:10.1038/onc.2009.238

7. Subramanian A, Tamayo P, Mootha VK, Mukherjee S, Ebert BL, Gillette MA, Paulovich A, Pomeroy SL, Golub TR, Lander ES, et al. Gene set enrichment analysis: A knowledge-based approach for interpreting genome-wide expression profiles. *Proc Natl Acad Sci* (2005) doi:10.1073/pnas.0506580102

8. Braun DM, Chung I, Kepper N, Deeg KI, Rippe K. TelNet - a database for human and yeast genes involved in telomere maintenance. *BMC Genet* (2018) **19**:32. doi:10.1186/s12863-018-0617-8

9. Gerber T, Willscher E, Loeffler-Wirth H, Hopp L, Schadendorf D, Schartl M, Anderegg U, Camp G, Treutlein B, Binder H, et al. Mapping heterogeneity in patient-derived melanoma cultures by single-cell RNA-seq. *Oncotarget* (2017) **8**:846–862. doi:10.18632/oncotarget.13666

10. Pino MS, Chung DC. The Chromosomal Instability Pathway in Colon Cancer. *Gastroenterology* (2010) **138**:2059–2072. doi:10.1053/j.gastro.2009.12.065

11. Binder H, Hopp L, Lembcke K, Wirth H. *Personalized disease phenotypes from massive OMICS data*. (2014). doi:10.4018/978-1-4666-6611-5.ch015

12. Lee M, Hills M, Conomos D, Stutz MD, Dagg RA, Lau LMS, Reddel RR, Pickett HA. Telomere extension by telomerase and ALT generates variant repeats by mechanistically distinct processes. *Nucleic Acids Res* (2014) **42**:1733–46. doi:10.1093/nar/gkt1117

13. Forsythe HL, Jarvis JL, Turner JW, Elmore LW, Holt SE. Stable Association of hsp90 and p23, but Not hsp70, with Active Human Telomerase. *J Biol Chem* (2001) **276**:15571–15574. doi:10.1074/jbc.C100055200

14. Jeong SA, Kim K, Lee JH, Cha JS, Khadka P, Cho H-S, Chung IK. Akt-mediated phosphorylation increases the binding affinity of hTERT for importin to promote nuclear translocation. *J Cell Sci* (2015) **128**:2951–2951. doi:10.1242/jcs.176453

15. Frohnert C, Hutten S, Wälde S, Nath A, Kehlenbach RH. Importin 7 and Nup358 Promote Nuclear Import of the Protein Component of Human Telomerase. *PLoS One* (2014) **9**:e88887. doi:10.1371/journal.pone.0088887

16. Cohen SB, Graham ME, Lovrecz GO, Bache N, Robinson PJ, Reddel RR. Protein Composition of Catalytically Active Human Telomerase from Immortal Cells. *Science (80-)* (2007) **315**:1850–1853. doi:10.1126/science.1138596

17. Tseng C-K, Wang H-F, Burns AM, Schroeder MR, Gaspari M, Baumann P. Human Telomerase RNA Processing and Quality Control. *Cell Rep* (2015) **13**:2232–2243. doi:10.1016/j.celrep.2015.10.075

18. Schmidt JC, Cech TR. Human telomerase: biogenesis, trafficking, recruitment, and activation. *Genes Dev* (2015) **29**:1095–1105. doi:10.1101/gad.263863.115

19. Majumder M, House R, Palanisamy N, Qie S, Day TA, Neskey D, Diehl JA, Palanisamy V. RNA-Binding Protein FXR1 Regulates p21 and TERC RNA to Bypass p53-Mediated Cellular Senescence in OSCC. *PLOS Genet* (2016) **12**:e1006306. doi:10.1371/journal.pgen.1006306

20. Pickett HA, Reddel RR. Molecular mechanisms of activity and derepression of alternative lengthening of telomeres. *Nat Struct Mol Biol* (2015) **22**:875–880. doi:10.1038/nsmb.3106

21. Cho NW, Dilley RL, Lampson MA, Greenberg RA. Interchromosomal Homology Searches Drive Directional ALT Telomere Movement and Synapsis. *Cell* (2014) **159**:108–121. doi:10.1016/j.cell.2014.08.030

22. Dilley RL, Verma P, Cho NW, Winters HD, Wondisford AR, Greenberg RA. Break-induced telomere synthesis underlies alternative telomere maintenance. *Nature* (2016) **539**:54–58. doi:10.1038/nature20099

23. Blasco MA. The epigenetic regulation of mammalian telomeres. *Nat Rev Genet* (2007) **8**:299–309. doi:10.1038/nrg2047

24. Conomos D, Reddel RR, Pickett HA. NuRD–ZNF827 recruitment to telomeres creates a molecular scaffold for homologous recombination. *Nat Struct Mol Biol* (2014) **21**:760–770. doi:10.1038/nsmb.2877

25. Zhong Z-H, Jiang W-Q, Cesare AJ, Neumann AA, Wadhwa R, Reddel RR. Disruption of Telomere Maintenance by Depletion of the MRE11/RAD50/NBS1 Complex in Cells That Use Alternative Lengthening of Telomeres. *J Biol Chem* (2007) **282**:29314–29322. doi:10.1074/jbc.M701413200

26. Jiang W-Q, Zhong Z-H, Henson JD, Neumann AA, Chang AC-M, Reddel RR. Suppression of Alternative Lengthening of Telomeres by Sp100-Mediated Sequestration of the MRE11/RAD50/NBS1 Complex. *Mol Cell Biol* (2005) **25**:2708–2721. doi:10.1128/MCB.25.7.2708-2721.2005

27. Grobelny J V, Godwin AK, Broccoli D. ALT-associated PML bodies are present in viable cells and are enriched in cells in the G(2)/M phase of the cell cycle. *J Cell Sci* (2000) **113 Pt 24**:4577–85. Available at: http://www.ncbi.nlm.nih.gov/pubmed/11082050 [Accessed July 12, 2019]

28. Wu G, Jiang X, Lee W-H, Chen P-L. Assembly of functional ALT-associated promyelocytic leukemia bodies requires Nijmegen Breakage Syndrome 1. *Cancer Res* (2003) **63**:2589–95. Available at: http://www.ncbi.nlm.nih.gov/pubmed/12750284 [Accessed July 12, 2019]

29. Jiang W-Q, Nguyen A, Cao Y, Chang AC-M, Reddel RR. HP1-Mediated Formation of Alternative Lengthening of Telomeres-Associated PML Bodies Requires HIRA but Not ASF1a. *PLoS One* (2011) **6**:e17036. doi:10.1371/journal.pone.0017036

30. Teasley DC, Parajuli S, Nguyen M, Moore HR, Alspach E, Lock YJ, Honaker Y, Saharia A, Piwnica-Worms H, Stewart SA. Flap Endonuclease 1 Limits Telomere Fragility on the Leading Strand. *J Biol Chem* (2015) **290**:15133–15145. doi:10.1074/jbc.M115.647388

31. Lovejoy CA, Li W, Reisenweber S, Thongthip S, Bruno J, de Lange T, De S, Petrini JHJ, Sung PA, Jasin M, et al. Loss of ATRX, Genome Instability, and an Altered DNA Damage Response Are Hallmarks of the Alternative Lengthening of Telomeres Pathway. *PLoS Genet* (2012) **8**:e1002772. doi:10.1371/journal.pgen.1002772

32. Lee M, Teber ET, Holmes O, Nones K, Patch A-M, Dagg RA, Lau LMS, Lee JH, Napier CE, Arthur JW, et al. Telomere sequence content can be used to determine ALT activity in tumours. *Nucleic Acids Res* (2018) **46**:4903–4918. doi:10.1093/nar/gky297

33. Min J, Wright WE, Shay JW. Alternative Lengthening of Telomeres Mediated by Mitotic DNA Synthesis Engages Break-Induced Replication Processes. *Mol Cell Biol* (2017) **37**: doi:10.1128/MCB.00226-17

34. Napier CE, Huschtscha LI, Harvey A, Bower K, Noble JR, Hendrickson EA, Reddel RR. ATRX represses alternative lengthening of telomeres. *Oncotarget* (2015) **6**:16543–58. doi:10.18632/oncotarget.3846
